# Genetic, Physiological, and Gene Expression Analyses Reveal That Multiple QTL Enhance Yield of Rice Mega-Variety IR64 under Drought

**DOI:** 10.1371/journal.pone.0062795

**Published:** 2013-05-08

**Authors:** Mallikarjuna Swamy B. P., Helal Uddin Ahmed, Amelia Henry, Ramil Mauleon, Shalabh Dixit, Prashant Vikram, Ram Tilatto, Satish B. Verulkar, Puvvada Perraju, Nimai P. Mandal, Mukund Variar, Robin S., Ranganath Chandrababu, Onkar N. Singh, Jawaharlal L. Dwivedi, Sankar Prasad Das, Krishna K. Mishra, Ram B. Yadaw, Tamal Lata Aditya, Biswajit Karmakar, Kouji Satoh, Ali Moumeni, Shoshi Kikuchi, Hei Leung, Arvind Kumar

**Affiliations:** 1 International Rice Research Institute (IRRI), Los Baños, Laguna, Philippines; 2 Directorate of Rice Research (DRR), Hyderabad, Andhra Pradesh, India; 3 Indira Gandhi Krishi Vishwavidyalaya (IGKV), Raipur, Chattishgarh, India; 4 Jawaharlal Nehru Krishi Vishwavidyalaya (JNKVV), Jabalpur, Madhya Pradesh, India; 5 Central Rainfed Upland Rice Research Station (CRURRS), Hazaribagh, India; 6 Tamil Nadu Agricultural University (TNAU), Coimbatore, Tamil Nadu, India; 7 Central Rice Research Institute (CRRI), Cuttack, India; 8 Narendra Dev University of Agriculture and Technology (NDUAT), Faizabad, Uttar Pradesh, India; 9 ICAR Research Complex for NEH Region, Tripura Centre (ICARNEHR), Tripura, India; 10 Regional Agricultural Research Station (RARS), Nepalganj, Nepal; 11 National Rice Research Program, Hardinath, Nepal; 12 Bangladesh Rice Research Institute (BRRI), Gazipur, Bangladesh; 13 Plant Genome Research Unit, Agrogenomics Research Center, National Institute of Agrobiological Sciences (NIAS), Tsukuba, Ibaraki, Japan; Cankiri Karatekin University, Turkey

## Abstract

**Background:**

Rice (*Oryza sativa* L.) is a highly drought sensitive crop, and most semi dwarf rice varieties suffer severe yield losses from reproductive stage drought stress. The genetic complexity of drought tolerance has deterred the identification of agronomically relevant quantitative trait loci (QTL) that can be deployed to improve rice yield under drought in rice. Convergent evidence from physiological characterization, genetic mapping, and multi-location field evaluation was used to address this challenge.

**Methodology/Principal Findings:**

Two pairs of backcross inbred lines (BILs) from a cross between drought-tolerant donor Aday Sel and high-yielding but drought-susceptible rice variety IR64 were produced. From six BC_4_F_3_ mapping populations produced by crossing the +QTL BILs with the −QTL BILs and IR64, four major-effect QTL - one each on chromosomes 2, 4, 9, and 10 - were identified. Meta-analysis of transcriptome data from the +QTL/−QTL BILs identified differentially expressed genes (DEGs) significantly associated with QTL on chromosomes 2, 4, 9, and 10. Physiological characterization of BILs showed increased water uptake ability under drought. The enrichment of DEGs associated with root traits points to differential regulation of root development and function as contributing to drought tolerance in these BILs. BC_4_F_3_-derived lines with the QTL conferred yield advantages of 528 to 1875 kg ha^−1^ over IR64 under reproductive-stage drought stress in the targeted ecosystems of South Asia.

**Conclusions/Significance:**

Given the importance of rice in daily food consumption and the popularity of IR64, the BC_4_F_3_ lines with multiple QTL could provide higher livelihood security to farmers in drought-prone environments. Candidate genes were shortlisted for further characterization to confirm their role in drought tolerance. Differential yield advantages of different combinations of the four QTL reported here indicate that future research should include optimizing QTL combinations in different genetic backgrounds to maximize yield advantage under drought.

## Introduction

Among cereals, rice (*Oryza sativa* L.) is the most drought-sensitive crop. Even a mild drought stress during the reproductive stage results in severe yield losses [1]–[3]. Most of the semi-dwarf high-yielding varieties developed during the green revolution era were meant for irrigated ecosystems and are highly susceptible to drought [4]. Since high-yielding drought-tolerant cultivars are not available, farmers in drought-prone areas cultivate either high-yielding cultivars with good grain quality that are drought susceptible or low-yielding traditional cultivars that are drought tolerant but have poor grain quality and also less input-use efficiency [5]–[7].

An understanding of the sources of genetic variation and physiological mechanisms involved facilitates the development of an appropriate strategy to breed drought-tolerant cultivars [8], [9]. Deep root growth, which may increase water uptake during progressive soil drying, is suggested to be a likely mechanism to confer increased yield under drought. However, there is little direct evidence in the literature of deep root growth conferring a yield advantage under drought [10]. A drought-yield effect of QTLs for deep roots and improved soil penetration [11]–[14] is yet to be confirmed.

Recent studies have identified QTL for yield under drought in rice [15]–[18]. Some of these QTL were derived from traditional donors and carry linkages for undesirable traits along with an effect on grain yield under drought [18]. The advanced backcross QTL (AB-QTL) approach involves two or more backcrosses to the improved recurrent parent to simultaneously identify and introgress QTL in the recurrent parent and to reduce undesirable linkages [19], [20]. AB-QTL analysis on lines with similar agro-morphological characters also provides the opportunity to impose uniform drought stress on all lines and to control differences due to phenology, leading to the detection of more reliable QTL. However, the genetic mapping of complex traits from parents with similar genetic backgrounds is difficult due to low polymorphism.

Expression profiling of contrasting parents under drought stress helps to identify differentially expressed genes and their regions in the genome [21]. The regions enriched with differentially expressed genes can be further genotyped with polymorphic molecular markers to detect the loci for complex traits. The differential expression patterns of drought-responsive genes in different plant tissues at different growth stages could provide an opportunity to characterize the traits associated with yield advantage under drought and to understand the physiological and molecular mechanisms that confer increased drought tolerance.

In this study, major QTL for grain yield under drought were narrowly delimited by expression polymorphism, and then identified in multiple mapping populations by genotyping and phenotyping under managed drought stress. We report physiological differences in backcross inbred lines (BILs) that were genetically similar but showed contrasting responses in yield under drought. The study identified lines with different combinations of QTL in the IR64 background that showed enhanced grain yield under drought in multi-location evaluations in the target environment, thereby confirming the value of these QTL for sustainable yield under drought stress.

## Results

### Four QTL for Grain Yield under Drought Identified

To define the QTL regions responsible for improved grain yield under drought in BILs derived from and IR64**×**Aday Sel cross [22] (Table S1), we used Affymetrix Rice Chip analysis to identify genome polymorphism. This approach was chosen after attempts to characterize the QTL regions with SSR markers did not reveal sufficient polymorphism between the parents. Four polymorphic regions were found at 6.8–7.3, 6.7–7.2, 14.6–16.5, and 18.6–19.3 Mb on chromosomes 2, 4, 9, and 10, respectively. In total, 5, 3, 8, and 5 polymorphic SSR markers in the regions detected by the chip-based analysis on chromosomes 2, 4, 9, and 10, respectively, were run on the whole population to detect QTL for grain yield (GY) (Table 1) and related traits (days to 50% flowering, DTF; plant height, PH; Table S2). The mapping results validated the regions predicted by the Affymetrix Rice Chip analysis. Four QTL were identified for GY under drought stress; *qDTY_2.2_*, *qDTY_4.1_*, *qDTY_9.1_,* and *qDTY_10.1_*, by both Interval and Composite Interval mapping methods (Table 1 and Fig. S1). The phenotypic variance explained by the QTL ranged from 6 to 19.2% (Table 1). None of these four *qDTY* QTL was associated with GY under non-stress conditions. QTL analyses in connected populations also consistently detected the major effect QTL *qDTY_2.2_* and *qDTY_9.1_* (Table S3 and Fig. S2).

**Table 1:** QTL for grain yield under drought in IR64**×**Aday Sel derived populations.

Population	Year	Chromosome	Marker interval	Peak marker	LOD	F value	R^2^	Additive effect
IR77298-5-6-B-18/IR64 (P1)	DS09	9	RM566-RM24350	RM566	5.5	39.4	9.6	201.7
IR77298-5-6-B-18/IR64 (P1)	DS10	9	RM566-RM24350	RM566	4.7	36.7	8.2	89.2
IR77298-5-6-B-18/IR64 (P1)	DS09&DS10	9	RM566-RM24350	RM566	7.6	50.9	13.0	134.4
IR77298-5-6-B-18/IR77298-5-6-B-11(P2)	DS09	9	RM566-RM24350	RM566	11.7	77.2	19.0	352.6
IR77298-5-6-B-18/IR64 (P1)	DS09	2	RM236-279	RM236	3.4	28.6	6.0	166.7
IR77298-5-6-B-18/IR64 (P1)	DS10	2	RM236-RM279	RM236	5.4	19.5	9.3	105.5
IR77298-5-6-B-18/IR64 (P1)	DS09&DS10	2	RM236-RM279	RM236	5.3	37.3	9.1	121.8
IR77298-14-1-2/IR64 (P4)	DS08	2	RM236/RM279-RM555	RM236/RM279	6.5	35.0	11.2	112.8
IR77298-14-1-2-B-10/IR64 (P3)	DS10	2	RM236-RM279	RM236	1.47	108.3	3.0	147.5
IR77298-14-1-2-B-10/IR64 (P3)	DS10	10	RM258-RM25694	RM258	10.0	28.7	17.0	298.1
IR77298-14-1-2/IR64 (P4)	WS07	4	RM335-RM518	RM518	6.5	14.7	11.2	127.1

The allelic source for all QTL was Aday Sel. LOD, Logarithm of odds ratios; R^2^, Phenotypic variance; Additive effect, grain yield (kg ha^−1^) additive effect over the population mean presented in Table S1.

### Physiological Characterization of BILs Contrasting for Yield under Drought

**Figure 1:**
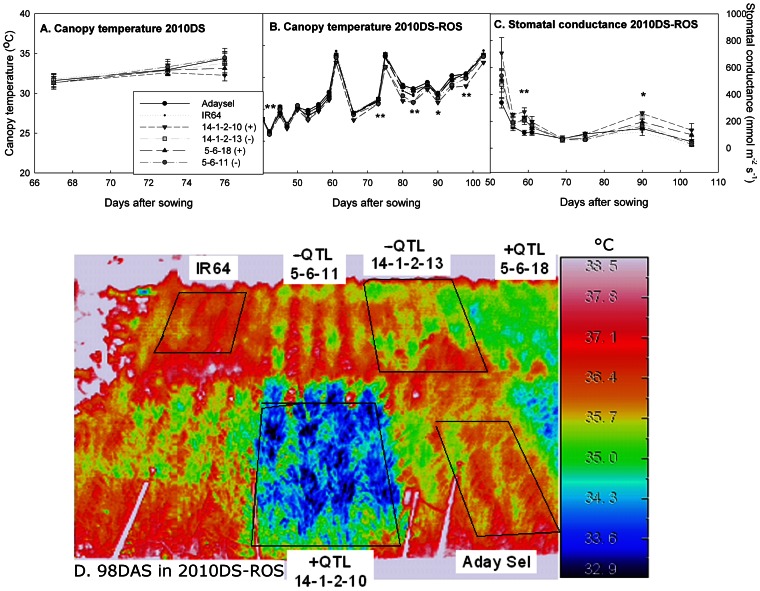
Canopy temperature dynamics over the A. 2010DS and B. 2010DS-ROS, as measured mid-day on sunny days with an infrared camera, and C. stomatal conductance during the 2010DS-ROS. Significant differences among lines are indicated by *(p<0.05) and **(p<0.01). D. Infrared thermal image showing contrasting canopy temperatures of +QTL and –QTL lines, taken 98 days after sowing (DAS) in 2010DS-ROS.

**Figure 2:**
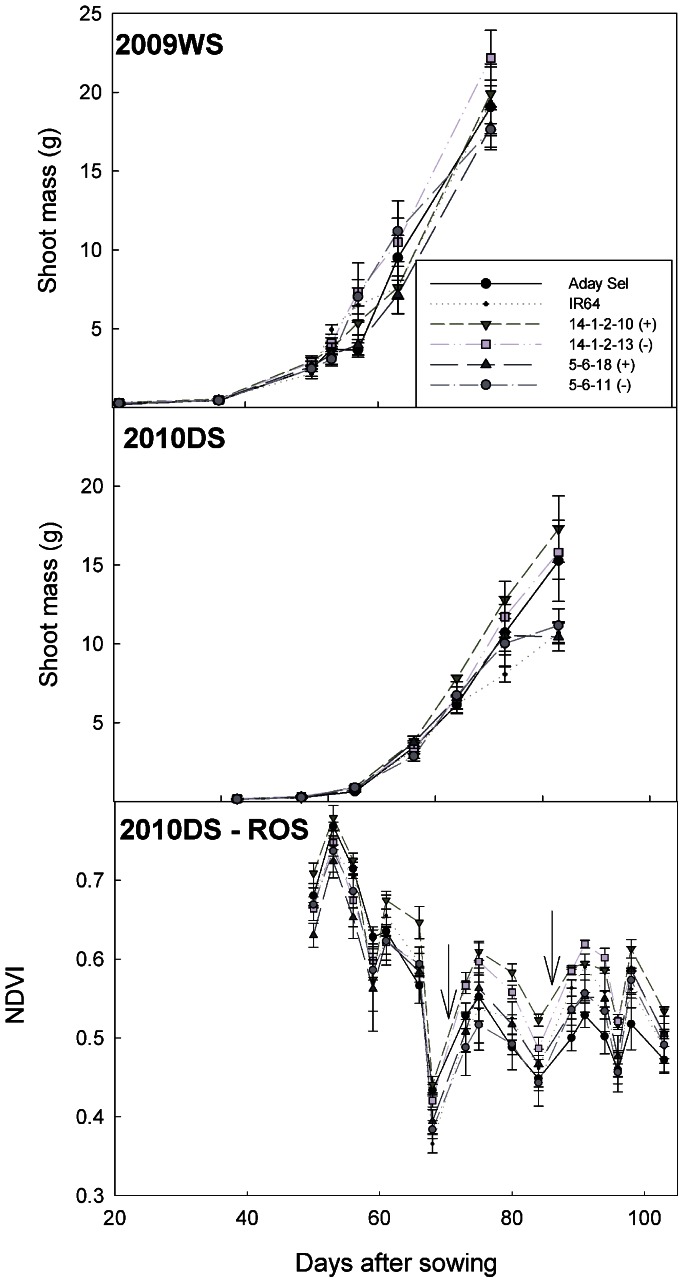
Shoot growth dynamics in field studies as measured by destructive sampling in 2009WS and 2010DS seasons, and by NDVI in 2010DS-ROS. Arrows indicate dates that the experiment was re-watered. No significant differences were observed between lines within a BIL pair on any sampling date.

Two pairs of genetically similar BILs (+QTL and –QTL lines from an IR64 **×** Aday Sel cross; pair 1: +QTL IR77298-5-6-B-18 and −QTL IR77298-5-6-B-11, and pair 2: +QTL IR77298-14-1-2-B-10 and –QTL IR77298-14-1-2-B-13) that were contrasting for yield under drought were characterized in the field to study the physiological mechanisms associated with increased yield under drought. +QTL lines showed cooler canopy temperature and greater stomatal conductance than −QTL lines and IR64 under the most severe drought stress, but not under mild drought stress or non-stress conditions (Fig. 1, Fig. S3). BILs within each pair did not differ significantly in shoot mass or NDVI (Normalized Difference Vegetation Index; Fig. 2), but shoot mass was greater towards the end of the 2010DS and NDVI was consistently greater in 2010DS-ROS in one pair of BILs (+QTL IR77298-14-1-2-B-10 and –QTL IR77298-14-1-2-B-13) compared to the other pair. Root growth at depth in terms of root length density was not greater in +QTL lines in any experiment (Table 2).

**Table 2:** Root length density of +QTL lines, −QTL lines, and IR64 at different depths in field drought studies, as sampled with a 4-cm diameter corer tube placed mid-way between hills of adjacent rows.

Root Length Density (cm cm^−3^)				
Depth	Genotype	2009 WS	2010DS	2010DS-ROS
**0–15 cm**	**IR64**	7.07±0.96	9.24±1.09	0.89±0.02
	**IR77298-14-1-2-B-10 (+)**			0.80±0.12
	**IR77298-14-1-2-B-13 (−)**			0.79±0.16
	**IR 77298-5-6-B-18 (+)**	4.80±0.48	12.91±2.48	0.63±0.03
	**IR77298-5-6-B-11(−)**	8.36±1.86	11.43±1.45	1.00±0.14
**15–30 cm**	**IR64**	2.95±0.52	3.58±0.61	0.75±0.09
	**IR77298-14-1-2-B-10 (+)**			0.74±0.08
	**IR77298-14-1-2-B-13 (−)**			0.86±0.19
	**IR 77298-5-6-B-18 (+)**	2.64±0.35	3.04±0.57	0.74±0.14
	**IR77298-5-6-B-11(−)**	2.33±0.22	2.67±0.70	0.92±0.09
**30–45 cm**	**IR64**	0.69±0.11	1.16±0.20	0.41±0.15
	**IR77298-14-1-2-B-10 (+)**			0.36±0.05
	**IR77298-14-1-2-B-13 (−)**			0.49±0.06
	**IR 77298-5-6-B-18 (+)**	0.55±0.07	0.99±0.17	0.48±0.26
	**IR77298-5-6-B-11(−)**	0.48±0.06	1.44±0.63	0.52±0.10
**45–60 cm**	**IR64**	0.29±0.07	0.69±0.16	0.49±0.16
	**IR77298-14-1-2-B-10 (+)**			0.52±0.16
	**IR77298-14-1-2-B-13 (−)**			0.65±0.13
	**IR 77298-5-6-B-18 (+)**	0.32±0.08	0.79±0.14	0.19±0.10
	**IR77298-5-6-B-11(−)**	0.33±0.06	1.08±0.42	0.54±0.23

Values shown are means ± s.e. No significant differences were observed among genotypes at any depth sampled.

### Meta-analysis of Differentially Expressed Genes Relative to the Entire Genome and within the Drought Yield QTL Regions

Transcriptome data of the same parental +QTL and –QTL BILs under two water stress treatments (0.5 FTSW, 0.2 FTSW) from a previous study by Moumeni *et al.* [21] were re-analyzed to determine their association with QTL detected in this study. For the root transcriptome, highly contrasting counts of DEGs were detected from comparison of +QTL and –QTL lines between the two BIL pairs used, with 570 DEGs in pair 1 and 2,127 DEGs in pair 2, an almost fourfold difference in DEG counts between the two BIL pairs. For the leaf transcriptome, a similar number of DEGs was detected from comparison of +QTL and –QTL lines between the two BIL pairs (748 DEGs in pair 1; 779 in pair 2). For the panicle transcriptome, both +QTL and –QTL BIL pairs had similar but low DEG counts (240 DEGs in pair 1; 201 in pair 2).

For the first meta-analysis, the number of DEGs was counted within 1 MB genome blocks, with sliding window blocks of 500 kb, for the entire genome, to determine whether DEGs were aggregating (having a significantly higher number of DEGs than anywhere else in the genome at p<0.01) in blocks of the genome, adapting the genomic method of Bruce *et al.* [23] for gene expression data. For pair 1, aggregation analysis of the root, leaf, and panicle transcriptomes all pointed to the five genome regions in chromosomes 5, 9, 10, and 12. For pair 2, transcriptomes from leaf and panicle tissues showed five overlapping regions of DEG aggregates in chromosomes 2, 8, and 11, whereas, for the root transcriptome, only the chromosome 8 DEG aggregation region overlapped with the regions from leaf and panicle tissues, and a unique aggregation region was found in chromosome 5. A total of 5 and 9 distinct DEG aggregation regions were determined for pairs 1 and 2, respectively, and no common regions exist between the two BIL pairs (Table S4).

The second meta-analysis used QTL region information from this study. Using all the DEGs from the significance (re)analysis, we found that there was significant association of 96 DEGs in QTL on chromosomes 2, 4, 9, and 10 (p<0.05, Table 3), with 91 of these DEGs located within the DEG aggregation regions found in chromosomes 2, 9, and 10. Further analysis for enrichment of biological themes/categories in this subset of DEGs, however, showed no explicitly drought-responsive categories being identified using the GO-SLIM and Mapman gene classification (Table 4). However, interesting significant associations of these DEGs with previously identified QTL (p<0.05, using the Gramene QTL categories) were found, such as QTL for root length, deep root dry weight, root weight, penetrated root thickness, and root penetration index, as well as QTL associated with yield traits (spikelet density, spikelet fertility, seed weight, and other grain/seed-related QTL; Table S5).

**Table 3:** Number of differentially expressed genes in +QTL and –QTL lines under two drought stress conditions.

+QTL NIL	Transcriptomesource tissue	AssociatedQTL	Number of DEGswithin QTL	Enrichment of DEGs within QTLregions (Fisher exact test p value)
IR77298-5-6-B-18	leaf	DTY 10.1	15	0.003
		DTY 9.1	23	0.007
				
	panicle	DTY 10.1	7	0.004
				
	root	DTY 10.1*	19	0.029
		DTY 10.1*	11	0.045
				
IR77298-14-1-2-B-10	leaf	DTY 2.2	8	0.000
				
	root	DTY 2.2	6	0.025
		DTY 4.1	5	0.032

DEG, differentially expressed genes; *DTY 10.1 resolves to two adjacent regions when physically mapped to the Nipponbare reference genome.

**Table 4:** Enriched GO-SLIM and Mapman pathways from the differentially expressed genes associated with drought-yield QTL.

Biologicalcategory	Gene category	Number ofassociated DEGs	Enrichmentp value
GO-SLIM_TG5	biological_process|amino acid and derivative metabolic process	9	0.0144
	cellular_component|plastid	9	0.0315
	cellular_component|thylakoid	8	0.0150
	molecular_function|lipid binding	4	0.0320
	molecular_function|molecular_function	11	0.0304
	molecular_function|oxygen binding	8	0.0007
	molecular_function|transferase activity	18	0.0007
Mapman release 31	amino acid metabolism.synthesis.serine-glycine-cysteine group.cysteine.OASTL	1	0.0497
	glycolysis.PPFK	1	0.0334
	misc.misc2	3	0.0005
	misc.protease inhibitor/seed storage/lipid transfer protein (LTP) family protein	4	0.0001
	misc.UDP glucosyl and glucoronyl transferases	5	0.0054
	not assigned.no ontology.pentatricopeptide (PPR) repeat-containing protein	12	0.0000
	polyamine metabolism.synthesis.SAM decarboxylase	1	0.0235
	protein.aa activation. valine-tRNA ligase	1	0.0169
	protein.degradation.serine protease	3	0.0105
	protein.glycosylation	3	0.0009
	protein.synthesis.initiation.deoxyhypusine synthase	1	0.0068
	secondary metabolism.flavonoids.dihydroflavonols.flavonoid 3′-monooxygenase	1	0.0068
	secondary metabolism.phenylpropanoids.lignin biosynthesis.HCT	1	0.0465

### Improved Lines with QTL Introgressions Identified

From the six mapping populations, 4 lines with four QTL combinations, 15 lines with three QTL combinations, 29 lines with two QTL combinations, and 19 lines with different single QTL were identified. These lines were further selected for yield under drought and under well-watered conditions, high phenotypic and genetic similarity to IR64, and grain quality traits similar to those of IR64 (Table 5, Table S6). Before advancing to evaluation in the target environment, the IR64 QTL lines were screened under managed drought stress in large plots at IRRI, in which all lines showed a yield advantage of 194 to 1920 kg ha^−1^ over IR64. Subsequently, the three most promising lines, IR87707-445-B-B, IR87707-446-B-B, and IR87707-182-B-B, were evaluated in target drought-prone ecosystems in Bangladesh, India, and Nepal. The three lines showed yield advantages of 528 to 1875 kg ha^−1^ over IR64 under drought, and produced either similar or higher yields than IR64 under well-watered conditions (Table 6).

**Table 5:** Yield and quality traits of IR64 QTL-introgressed lines at IRRI.

Line	QTL	DTF (NS)	PH (cm) (NS)	GY (kg ha^−1^) (NS)		GY (kg ha^−1^) (S)		Bio (kg ha^−1^) NS	Bio (kg ha^−1^) S	AC (%)	GT	MP	CS	GS(%)
		DS11	DS11	DS10	DS11	DS10	DS11	DS12	DS12					
IR87729-69-B-B-B	*qDTY_9.1_, qDTY_2.2_, qDTY_10.1_, qDTY_4.1_*	83	91	4312	6308	2011	1943	5541	4007	20.7	I	1	1	94.4
IR87728-491-B-B	*qDTY_9.1_, qDTY_2.2_, qDTY_4.1_*	82	95		6232	1041	1879	5255	3557	20.3	I	1	1	92.6
IR87707-186-B-B-B	*qDTY_2.2_, qDTY_10.1_, qDTY_4.1_*	78	99	4550	6103	2068	2632	4865	3759	21.6	I	2	1	96.9
IR87707-359-B-B-B	*qDTY_2.2_, qDTY_10.1_, qDTY_4.1_*	81	98	4638	6361	1934	2581			22.0	I	2	2	95.3
IR87707-446-B-B-B	*qDTY_2.2_, qDTY_4.1_*	80	98	3752	4388	2556	3000	5591	3811	22.2	I	1	1	97.0
IR87707-445-B-B-B	*qDTY_2.2_, qDTY_4.1_*	77	96	5045	5844	2555	3023	5347	3824	22.3	I	1	1	96.9
IR 87707-182-B-B-B	*qDTY_2.2_, qDTY_4.1_*	78	97	3875	5225	1926	2891			22.1	I	1	1	96.9
IR87728-162-B-B	*qDTY_9.1_, qDTY_2.2_*	84	94		6115	1147	1636	5642	3445	20.1	I	1	1	92.4
IR87705-83-12-B	*qDTY_2.2_, qDTY_10.1_*	80	95	4796	5526	1916	2270	4946	3366	19.8	I	1	1	95.0
IR87705-80-15-B	*qDTY_10.1_, qDTY_4.1_*	81	89	3850	5516	2074	2151			17.8	I	2	1	94.6
IR87705-72-12-B	*qDTY_2.2_*	80	91	3569	6090	1879	1892	6064	3661	19.2	I	2	1	96.5
IR87705-6-8-B	*qDTY_4.1_*	81	88	5399	6208	2152	2588			21.0	I/L	2	1	95.5
IR87728-395-B-B	*qDTY_9.1_*	83	92		6627	2440	2046	4608	3762	19.1	I	2	1	93.4
IR87705-36-3-B	*qDTY_10.1_*	82	97	5052	6909		2116	6047	3501	20.3	I	1	1	95.3
IR64		80	96	2987	5435	636	1442	4860	3015	21.8	I/L	1	1	
LSD 0.05		3	7		1053		690							

DTF, Days to 50% flowering; PH, Plant height; GY, Grain yield; Bio, Straw biomass at harvest; S, Stress; NS, Non-stress; AC, Amylose content; GT, Gelatinization temperature (I, intermediate; L, low); MP, Milling potential; CS, Chalkiness score; GS, Genetic similarity.

**Table 6:** Yield of QTL-introgressed IR64 lines in the target ecosystem in Bangladesh, Nepal, and India.

	Grain yield (kg ha^−1^)													
	Rajshahi		Nepalganj		Raipur		Hyderabad 1		Hyderabad 2		Hazaribagh		Rewa	
Entry	Non-stress	Drought stress	Non-stress	Drought stress	Non-stress	Drought stress	Non-stress	Drought stress	Non-stress	Drought stress	Non-stress	Drought stress	Non-stress	Drought stress
IR87707-445-B-B	4167	1525	5990	3472	5084	3956	5672	1684	5672	3800	5690	1604	4100	3731
IR87707-446-B-B	4521	1933	6302	2847	5771	3614	5634	1813	5634	4057	5711	1229	4911	3899
IR87707-182-B-B	4312	1508	6510	2778	5646	3419	6047	1383	6047	3604	4453	1500	4513	3509
IR64	3379	980	4297	1597	4083	2662	5699	660	5699	3085	5612	958	3586	2503
LSD	240	156	324	170	434	285	186	939	186	600	475	422	920	909

## Discussion

Crop genetic improvement for drought is challenging because of its complex genetic nature and poor understanding of the physiological and molecular mechanisms associated with drought tolerance [8], [9]. We applied multiple approaches including genetic mapping, physiological characterization, and expression analyses to identify major-effect drought grain yield QTL, and successfully deployed them to improve grain yield under drought in the background of rice mega-variety IR64.

QTL, when mapped back to the physical genome, often translate to tens of megabases in size, which is not precise for identification of gene(s) underlying the QTL effect. We used a meta-analysis approach that combines results from the analysis of transcriptome data (aggregation of DEGs within QTL, and gene set enrichment tests) and QTL position in the physical genome to overcome low resolution in genetic mapping and enable us to identify a smaller set of candidate genes within the QTL region.

The consistent effect of four drought-yield QTL in the background of popular variety IR64 indicates their suitability for marker-assisted breeding (MAB) to improve the drought tolerance of IR64. The region on chromosome 2 in which *qDTY_2.2_* was detected has been reported previously to have an effect on drought-related traits other than GY, including drought tolerance index, canopy temperature, osmotic adjustment, and leaf water content [24]–[27]. The clustering of a number of physiological traits in the same region suggests its importance for GY under severe drought stress. Differential expression of *qDTY_4.1_* was observed under different severities of stress, indicating its usefulness over a wide range of stress severities. Lanceras *et al.* [28] also reported that different QTL were detected on different chromosomes depending on the severity of stress, and that three different grain yield QTL on chromosome 4 were identified in the CT9993/IR62266 doubled-haploid (DH) population under non-stress, mild, and severe drought stress conditions. QTL for GY and for grains per panicle were also detected on chromosome 4 under drought stress conditions in the CT9993/IR62266 DH population at the distal end of chromosome 4 [29] and for spikelet fertility and for grain weight in the Bala/Azucena DH population [30]. *qDTY_9.1_* showed high phenotypic variance in both years, indicating that this QTL was equally effective under both severe and moderate drought stress. This region on chromosome 9 was reported to have a QTL for spikelet fertility (27), GY, and plant height under non-stress conditions [31], and for grain weight under non-stress conditions [32]–[34]. In joint analyses of connected populations, the major effect QTL *qDTY_2.2_* and *qDTY_9.1_* were also detected, indicating their reliability and worthiness for use in MAS. *qDTY_10.1_* with high additive effect and a high phenotypic variance of 17% was identified under severe drought stress in the population P3 in 2010DS. A QTL for GY was also reported in a nearby region on chromosome 10 under severe drought stress [25], [28], [29] and under non-stress conditions [35]. A QTL for grain weight was also found near *qDTY_10.1_* [36] under stress conditions. In all of these studies, including the present study, the yield-increasing allele was contributed by the drought-tolerant parent.

The physiological characterization of the BILs showed that +QTL lines maintained higher transpiration rates under drought, as evidenced by cooler canopy temperature and higher stomatal conductance than −QTL lines as soil water availability decreased (Fig. 1). We expected that this transpiration advantage in +QTL lines would be conferred by greater root growth at depth, but this was not observed. The similar shoot and root growth among +QTL and −QTL lines within BIL pairs suggests that the yield advantages under drought are not due to architectural or allometric differences in plant growth. Therefore, these results point to differences in water uptake among QTL lines; the QTL effect in the BILs appears to be associated with root anatomy/development or regulation of root function rather than deep root growth.

Gene expression in leaf, panicle, and root tissues under different drought stresses also pointed to differences in root development between the +QTL and −QTL BILs. Aggregation analysis of transcriptome data already available from genome-wide comparisons [21] allowed us to identify a subset of DEGs that aggregated in 14 blocks in the genome, which we then overlayed with QTL information independently generated in the present study, as well as from other published studies. The co-localization of DEGs in this study with published QTL suggests that some of the DEGs could be related to root structure and yield parameters. The convergent analysis of QTL, expression, and previously published data has enabled us to nominate a relatively small number of candidate genes for further investigation (Table S7). A caveat is that this analysis relied on a japonica reference genome, which may not contain the actual genes that are related to the QTL found. The availability of an IR64 reference genome and genome sequences for the +QTL BILs will provide more accurate positions of QTL and DEGs and is likely to improve the power of QTL enrichment analysis.

A literature survey was carried out to know the specific/broad biological functions of the differentially expressed genes/gene families between the +QTL and –QTL lines. In terms of gene function, most of the DEGs were associated with growth and development processes, stress responses and signaling, and hormonal regulation (Table S8). The growth- and development-related genes/gene families were associated with meristematic tissue activity in the roots, inflorescence, and leaf; genes for cell wall synthesis; root elongation; formation of lateral roots; pollen fertility; embryogenesis; seed development; and seed germination. The genes for hormonal regulation were associated with auxin and cytokinin homeostasis, the two most important hormones involved in growth and development processes. Many stress-signaling and stress-responsive genes were differentially expressed between the +QTL and −QTL lines, and an interesting abundance of four transcription factor genes all on chromosome 9 was observed (Os09 g24660: zinc finger motif, C2HC5-type family protein, putative, expressed; Os09 g24800: myb-related protein Myb4, putative; Os09 g25420: zinc finger, C2H2-type family protein, expressed; and Os09 g26180: transcription initiation factor TFIID subunit 10, putative, expressed). Although these expressed genes show some consistencies with the physiological responses observed at the whole-plant level in the field, further research is necessary to dissect the water uptake attributes associated with low canopy temperature under drought in these lines, as well as to identify the key genes within the QTL regions associated with drought response.

The higher yield of IR64+QTL NILs compared with that of IR64 under drought stress in two seasons indicates the consistent effect of the QTL. Among the lines with individual QTL, *qDTY_4.1_* lines performed better than lines with other QTL. Three lines with *qDTY_2.2_*, *qDTY_4.1_*, and *qDTY_10.1_*, and two lines with *qDTY_2.2_* and *qDTY_4.1_* QTL combinations performed better than lines with other QTL combinations, indicating a differential synergistic relationship between QTL combinations [37]. A better understanding of the complementary effects among the four QTL could enable pyramiding of QTL in appropriate combinations to maximize the yield advantage under drought. Such optimization of QTL combinations may be required for introgression into different genetic backgrounds. The best-performing IR64 QTL lines at IRRI were also the highest performing lines in Bangladesh, India, and Nepal, indicating stable performance of the IR64 QTL lines across environments and that results from managed dry-season drought screening at IRRI are relevant to the target drought-prone environments.

In this study, IR64 QTL lines were developed that confer a yield advantage of 500 to 1800 kg ha^−1^ under different severities of drought over IR64, a popular rice mega-variety grown on millions of hectares in Asia. These lines can be immediately disseminated for cultivation by farmers in drought-prone environments. The identification of multiple QTL that collectively enhance yield under drought in rice confirms the effectiveness of direct selection for increased yield under drought. Although canopy temperature and transpiration results indicated increased water uptake under drought, we did not see differences in root length density in the BILs, suggesting a role of the QTL in root function rather than root architecture. Hypotheses on the functional roles of these QTL were provided by convergent analysis of mapping and gene expression patterns, with candidate genes significantly enriched in the mapped QTL regions being nominated for further characterization. Differential yield advantages of different combinations of the four QTL reported here indicate that future research should include optimizing QTL combinations in different genetic backgrounds to maximize yield advantage under drought.

## Materials and Methods

### Plant Materials and Mapping Populations for Physiological and Genetic Study

Two BILs, IR77298-14-1-2 (BC_3_F_3∶4_) and IR77298-5-6-B-18 (BC_3_F_2∶3_), were derived from a cross between drought-tolerant traditional donor Aday Sel and drought-susceptible recurrent parent IR64. These backcross lines had been fixed for most of the agro-morphological traits such as days to 50% flowering (DTF), plant height (PH), grain yield (GY), and biomass (BIO), but showed differential performance under drought [22]. A set of around 60 single panicles from the two lines with differential performance under drought was selected and evaluated under drought stress in lowland conditions. For physiological characterization, we used two pairs of +QTL and −QTL BILs (IR77298-5-6-B-18 (+QTL) and IR77298-5-6-B-11 (−QTL) and IR77298-14-1-2-B-10 (+QTL), and IR77298-14-1-2-B-13 (−QTL) that showed contrasting performance for grain yield under drought. We presumed that the drought tolerant high yielding BILs contained QTL and drought susceptible low yielding BILs did not, and designated them as +QTL and –QTL lines respectively. Subsequently, +QTL lines (IR77298-5-6-B-18, IR77298-14-1-2-B-10) as well as IR77298-14-1-2, a drought tolerant line from the same population, were crossed with –QTL lines and with IR64 in different combinations to develop six mapping populations (Table S1) to identify, introgress, and pyramid QTL for grain yield under drought in the IR64 background.

### Phenotypic Characterization

All field-based phenotyping experiments were conducted at the International Rice Research Institute in lowland transplanted conditions (IRRI, Los Baños, Philippines, 14°30′N, 121°15′E). Drought stress (stress) and well-watered control (non-stress) experiments were managed as described by Venuprasad *et al.* [17].

To characterize the physiological response of +QTL and −QTL BILs, three lowland field experiments were conducted (2009 wet season (2009WS), 2010 dry season (2010DS), and 2010DS in a rainout shelter (2010DS-ROS). The soil was classified as an Isohyperthermic Typic Hapludalf in 2009WS and 2010DS, and Aquandic Epiaquall at the site used in 2010DS-ROS, ranging in bulk density at 30 cm from 0.86 to 1.3 g cm^−3^. Four replicates per genotype were planted in randomized compete block designs in plots of 3-m length with 4 rows per plot. Soil water potential was monitored in all seasons with three tensiometers installed in each experimental field (Soilmoisture Equipment Corp, CA, USA) and it reached −70 kPa at a depth of 20 cm from 60–75 days after sowing in 2010DS, indicating that the most severe drought treatment was applied in that season (Fig. S2). Canopy temperature was measured at mid-day from a 3-m ladder by infrared thermography (NEC Avio Infrared Technologies Co. Ltd., Tokyo, Japan). Leaf stomatal conductance was measured at mid-day with a porometer (AP4, Delta-T Devices, Cambridge, UK). Shoot growth dynamics were monitored by destructive harvests in 2009WS and 2010DS, and with NDVI measured around mid-day in 2010DS-ROS (Greenseeker Hand-held Sensor, NTech Industries, CA, USA). Root samples were taken at 94 DAS in 2009WS, at 73 DAS in 2010DS, and at 94 DAS in 2010DS-ROS to a depth of 60 cm using a 4-cm-diameter core sampler according to Henry *et al.* [38].

Six BC_4_F_3_ mapping populations were screened for grain yield under stress and non-stress conditions during the dry seasons (Table S1). An alpha lattice design with two replications and two 4-m rows per plot was used in all experiments. Based on cumulative rainfall, rainfall distribution, and yield reductions compared to the non-stress treatment, a range of drought severities was achieved. Severe droughts occurred in 2008DS, 2010DS, 2011DS, and 2012DS. In all experiments, data on DTF, PH, and GY per plot (normalized to 12% moisture content) were recorded as described in the standard evaluation system of IRRI [39].

### Defining QTL Regions using Affymetrix Rice Genome Array DNA Analysis

Based on screening with 600 SSR markers, low polymorphism was detected between the +QTL and –QTL BILs due to their similar genetic background. We used genomic DNA hybridization on an Affymetrix GeneChip® Rice Genome Array to determine regions with significant differences between BILs of one pair (IR77298-5-6-B-18 (+QTL) and IR77298-5-6-B-11 (−QTL)). Probeset intensity and differentially hybridized probesets (at p<0.05) between the +QTL and –QTL BILs were determined using the R/Bioconductor packages Affy and R/MAANOVA. To identify genome blocks that were polymorphic between the lines of interest, aggregation of differentially hybridized probesets along the genome was determined at 1000 kb, with sliding windows of 500 kb as described by Bruce *et al.* [23]. Out of 17 candidate regions, 4 regions that showed significant differences between the +QTL and –QTL BILs in the GeneChip results were selected for further QTL analysis. A polymorphism survey between parents of the six mapping populations was carried out on the four regions. A total of 15, 29, 24, and 33 markers in the four differentially expressed regions on chromosomes 2, 4, 9, and 10, respectively, were surveyed for polymorphism and we detected 3, 6, 4 and 5 polymorphic markers in those regions. Six populations, P1, P2, P3, P4, P5 and P6, with 487, 478, 455, 286, 485 and 488 individuals, respectively, were genotyped using the polymorphic markers and phenotyped for yield and yield-related traits under drought stress and non-stress conditions.

### Statistical Analysis

Statistical analysis was carried out using SAS v.9.1.3 [40] for the phenotypic data of mapping populations, and means of trait values were estimated using the REML algorithm of PROC MIXED. Statistical analyses for the physiology experiments were performed in R v. 2.8.0 [41] using ANOVA, and Tukey’s HSD was used for mean comparison.

### QTL Identification, and Differential Expressed Gene Analyses

QTL analysis was carried out following Interval mapping and Composite Interval Mapping (CIM) using Q gene software [42] and results were validated by QTL Cartographer [43]. A significance level of 0.01 was used to detect putative QTL. The minimal LOD value required to declare a QTL was obtained from 1000 permutation tests. QTL analyses were confirmed by performing CIM and iQTLM analysis in connected populations using MC-QTL software [44].

To identify differentially expressed genes within the four QTL regions, a reanalysis of the Agilent 44k oligoarray data was conducted using R/MAANOVA (significance p<0.001) to compare +QTL and –QTL BILs rather than drought treatments (FTSW 0.5 and 0.2; moderate and severe drought stress) as reported by Moumeni *et al*. [21]. The DEGs determined from the reanalysis were tested for enrichment in the four QTL regions (gene models from the Michigan State University Rice Genome Annotation Project, release 6.1: http://rice.plantbiology.msu.edu/) using a Fisher exact test (significance p<0.05, method from McNally *et al*. [45]. The same enrichment test was then made for all DEGs against the GO-SLIM, Mapman (http://mapman.gabipd.org/), and Gramene QTL (http://gramene.org/qtl/) annotation of the MSU 6.1 genome. All microarray data and information on the transcriptomes of the three tissues are available at NCBI GEO accessions GSE30463 (root expression data), GSE30449 (shoot expression data) and GSE30462 (panicle expression data) (http://www.ncbi.nlm.nih.gov/geo/).

### Identification of IR64 Introgression Lines

From among 2806 lines in six populations, a set of 84 lines with high yield under both stress and non-stress conditions, and with phenotypic similarity to IR64, was genotyped with foreground markers for QTL *qDTY_2.2_*, *qDTY_4.1_*, *qDTY_9.1_*, and *qDTY_10.1_* to identify lines with combinations of one, two, three, and four QTL. Background genotyping of the 84 lines was carried out with 580 SSR markers randomly distributed throughout the genome to identify introgression lines with high genetic similarity to IR64. In 2011DS, 84 lines were evaluated for yield under stress and non-stress conditions. These 84 lines were analyzed for amylose content (AC), gelatinization temperature (GT), milling potential (MP), and chalkiness (CS) following the protocol as described in the standard evaluation system of IRRI (39) with the aim to identify lines with quality traits similar to those of IR64. The three best identified IR64 QTL lines (Table 6) were evaluated under non-stress and drought stress conditions at one site each in Bangladesh (Rajshahi) and Nepal (Nepalganj) and at four sites in India (Hyderabad, Rewa, Raipur, and Hazaribagh).

## Supporting Information

Figure S1 **QTLs identified for grain yield under drought stress.**
(DOCX)

Figure S2 **Major effect QTL** ***DTY_2.2_*** **and** ***DTY_9.1_*** **identified in multiple populations.**
(DOCX)

Figure S3 **Soil water potential measured by tensiometers in each field study season.**
(DOCX)

Table S1 **Mean for yield and related traits under drought stress and non-stress conditions during 2007DS to 2010DS.**
(DOCX)

Table S2 **QTLs for yield related traits under drought stress and non-stress in IR64×Aday Sel derived populations.**
(DOCX)

Table S3 **QTLs for grain yield under drought identified by multi-population joint analyses.**
(DOCX)

Table S4 **Regions of aggregation of differentially expressed genes from the re-analyzed transcriptome dataset.**
(DOCX)

Table S5 **Enriched Gramene QTL categories of the subset candidate genes in IR77298-14-1-2-B-10 (+QTL) and IR77298-14-1-2-B-13 (−QTL).**
(DOCX)

Table S6 **Performance of 84 IR64-NILs under non –stress (NS) and drought stress (S) conditions.**
(DOCX)

Table S7 **List of candidate genes associated with yield-under-drought QTLs.**
(DOCX)

Table S8 **Candidate genes/gene families and their biological functions under stress conditions.**
(DOCX)

Text S1 **Supplementary References.**
(DOCX)
