# Supplementary figures and images for: Genetic, Physiological, and Gene Expression Analyses Reveal That Multiple QTL Enhance Yield of Rice Mega-Variety IR64 under Drought
Source: PLoS One. 2013 May 8;8(5):e62795. doi: 10.1371/journal.pone.0062795 (PMC3648568; doi:10.1371/journal.pone.0062795)

**
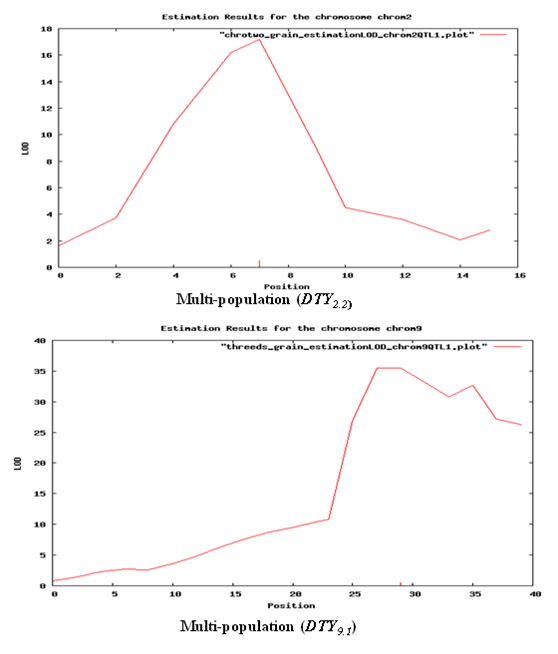
**

**Fig S2.**

Supplement: Figure S2 — Major effect QTL DTY2.2 and DTY9.1 identified in multiple populations. (DOCX) [file pone.0062795.s002.docx]
