# Supplementary material for: Genetic, Physiological, and Gene Expression Analyses Reveal That Multiple QTL Enhance Yield of Rice Mega-Variety IR64 under Drought
Source: PLoS One. 2013 May 8;8(5):e62795. doi: 10.1371/journal.pone.0062795 (PMC3648568; doi:10.1371/journal.pone.0062795)
Supplement: Table S1 — Mean for yield and related traits under drought stress and non-stress conditions during 2007DS to 2010DS. (DOCX) [file pone.0062795.s004.docx]

**Table S1.**

| **Population** | **Parentage** | **Season**  **tested** | **DTF** | | **GY(kg ha^-1^)** | |
| --- | --- | --- | --- | --- | --- | --- |
|  |  |  | **S** | **NS** | **S** | **NS** |
| P1 | IR77298-5-6-B-18/IR64  IR77298-5-6-B-18/IR64 | 2009DS  2010DS | 87  95 | 86  - | 1218  824 | 6073  - |
| P2 | IR77298-5-6-B-18/IR77298-5-6-B-11 | 2009DS | 87 | 85 | 1216 | 5510 |
| P3 | IR77298-14-1-2-B-10/IR64 | 2010DS | 90 | 85 | 1654 | 4520 |
| P4 | IR77298-14-1-2/ IR64  IR77298-14-1-2/IR64 | 2007WS  2008DS | 86  91 | 86  90 | 2011  1400 | 2556  4366 |
| P5 | IR77298-14-1-2-B-10/ IR77298-5-6-B-11 | 2009DS | 85 | 84 | 2094 | 6023 |
| P6 | IR77298-14-1-2-B-10 x IR77298-14-1-2-B-13 | 2009DS | 81 | 82 | 1929 | 5022 |
| **Parents** |  |  |  |  |  |  |
| IR64(09) P1 | IR5657-33-2-1/IR 2061-465-1-6-5 | 2009DS | 87 | 85 | 693 | 5064 |
| IR64(10) P2 | IR5657-33-2-1/IR 2061-465-1-6-5 | 2010DS | 99 | 85 | 542 | 4855 |
| IR64(10) P3 | IR5657-33-2-1/IR 2061-465-1-6-5 | 2010DS | 96 | 87 | 564 | 3394 |
| IR64(07)P4 | IR5657-33-2-1/IR 2061-465-1-6-5 | 2007WS | 87 | 87 | 703 | 3107 |
| IR64 (08)P4 | IR5657-33-2-1/IR 2061-465-1-6-5 | 2008DS | 91 | 91 | 993 | 2946 |
| IR77298-5-6-B-18(09) P1 | Aday Sel/IR64 | 2009DS | 84 | 85 | 1272 | 6140 |
| IR77298-5-6-B-18 (10) P1 | Aday Sel/IR64 | 2010DS | 92 | - | 1005 | - |
| IR77298-5-6-B-18(10) P2 | Aday Sel/IR64 | 2010DS | 87 | 87 | 1171 | 4436 |
| IR77298-5-6-B-11(09)P2 | Aday Sel/IR64 | 2009DS | 87 | 86 | 974 | 5340 |
| IR77298-5-6-B-11(10)P5 | Aday Sel/IR64 | 2010DS | 94 | 89 | 776 | 5421 |
| IR77298-14-1-2-B-10 (09)P3 | Aday Sel/IR64 | 2009DS | 79 | 83 | 1765 | 4755 |
| IR77298-14-1-2-B-10 (10)P5 | Aday Sel/IR64 | 2010DS | 85 | 83 | 1183 | 4192 |
| IR77298-14-1-2-B-10 (09)P6 | Aday Sel/IR64 | 2009DS | 81 | 80 | 2625 | 4837 |
| IR77298-14-1-2-1(07)P4 | Aday Sel/IR64 | 2007DS | 86 | 86 | 2730 | 3128 |
| IR77298-14-1-2-1 (08)P4 | Aday Sel/IR64 | 2008DS | 83 | 89 | 1805 | 3766 |
| IR77298-14-1-2-B-13 P6 | Aday Sel/IR64 | 2009DS | 81 | 82 | 1486 | 4476 |

DTF, Days to 50% flowering; GY, Grain yield; S, Stress; NS, Non-stress
