# Supplementary material for: Genetic, Physiological, and Gene Expression Analyses Reveal That Multiple QTL Enhance Yield of Rice Mega-Variety IR64 under Drought
Source: PLoS One. 2013 May 8;8(5):e62795. doi: 10.1371/journal.pone.0062795 (PMC3648568; doi:10.1371/journal.pone.0062795)
Supplement: Table S2 — QTLs for yield related traits under drought stress and non-stress in IR64×Aday Sel derived populations. (DOCX) [file pone.0062795.s005.docx]

| **Population** | **Traits** | **Season** | **Treatment** | **Chromosome** | **Marker interval** | **Peak marker** | **LOD** | **R^2^** | **Additive effect** | **Allelic source** |
| --- | --- | --- | --- | --- | --- | --- | --- | --- | --- | --- |
| P3 | DTF | DS10 | drought stress | 2 | RM236-RM279 | RM236 | 6.2** | 10.7 | -1.42 | IR64 |
| P4 | DTF | DS08 | drought stress | 2 | RM555-RM492 | RM555 | 5.6** | 9.8 | -0.71 | IR64 |
| P4 | PHT | DS08 | drought stress | 2 | RM555-RM492 | RM555 | 2.7** | 4.7 | 1.1 | Adaysel |
| P3 | DTF | DS10 | Non-stress | 2 | RM236-RM279 | RM236 | 5.7** | 10 | -0.27 | IR64 |
| P4 | DTF | DS08 | Non-stress | 2 | RM279-RM555 | RM279 | 3.5** | 6.2 | -0.41 | IR64 |

**Table S2.**
