# Supplementary material for: Genetic, Physiological, and Gene Expression Analyses Reveal That Multiple QTL Enhance Yield of Rice Mega-Variety IR64 under Drought
Source: PLoS One. 2013 May 8;8(5):e62795. doi: 10.1371/journal.pone.0062795 (PMC3648568; doi:10.1371/journal.pone.0062795)
Supplement: Table S3 — QTLs for grain yield under drought identified by multi-population joint analyses. (DOCX) [file pone.0062795.s006.docx]

**Table S3.**

| **Multi-population** | **chromosome** | **R2** |  |  | **Additive effect(Kg/ha)** | **Additive effect(Kg/ha)** |
| --- | --- | --- | --- | --- | --- | --- |
|  |  |  | **Allelic source** | **Recipient parent** | CIM | iQTLM |
| **P1, P3, P4** | 2 | 5 | IR77298-5-6-B-18 | IR64 | 84 | 84 |
|  |  |  | IR77298-14-1-2-B-10 | IR64 | 89 | 89 |
|  |  |  | IR77298-14-1-2 | IR64 | 41 | 41 |
| **P1, P2** | 9 | 11 | IR77298-5-6-B-18 | IR64 | 127 | 127 |
|  |  |  | IR77298-5-6-B-18 | IR77298-5-6-B-11 | 192 | 192 |
