# Supplementary material for: Genetic, Physiological, and Gene Expression Analyses Reveal That Multiple QTL Enhance Yield of Rice Mega-Variety IR64 under Drought
Source: PLoS One. 2013 May 8;8(5):e62795. doi: 10.1371/journal.pone.0062795 (PMC3648568; doi:10.1371/journal.pone.0062795)
Supplement: Table S4 — Regions of aggregation of differentially expressed genes from the re-analyzed transcriptome dataset. (DOCX) [file pone.0062795.s007.docx]

**Table S4.**

| **Transcriptome dataset** | **Aggregation region number** | **Chromosome** | **Start (bp)** | **End (bp)** |
| --- | --- | --- | --- | --- |
| IR77298-5-6-B-18 vs IR77298-5-6-B-11 : panicle |  |  |  |  |
|  | 1 | 5 | 26,184,282 | 29,670,091 |
|  | 2* | 9 | 14,693,433 | 16,647,821 |
|  | 3 | 10 | 11,649,622 | 12,646,013 |
|  | 4* | 10 | 18,091,107 | 19,586,024 |
|  | 5 | 12 | 22,496,653 | 24,481,038 |
| IR77298-5-6-B-18 vs IR77298-5-6-B-11 : leaf |  |  |  |  |
|  | 1 | 5 | 26,184,282 | 29,670,091 |
|  | 2* | 9 | 14,203,203 | 16,647,821 |
|  | 3 | 10 | 11,649,622 | 12,646,013 |
|  | 4* | 10 | 18,091,107 | 19,586,024 |
|  | 5 | 12 | 22,991,815 | 24,481,038 |
| IR77298-5-6-B-18 vs IR77298-5-6-B-11 : root |  |  |  |  |
|  | 1 | 5 | 26,184,282 | 29,670,091 |
|  | 2* | 9 | 14,203,203 | 16,647,821 |
|  | 3 | 10 | 11,153,736 | 13,136,168 |
|  | 4* | 10 | 17,594,205 | 20,082,285 |
|  | 5 | 12 | 22,991,815 | 24,481,038 |
| IR77298-14-1-2-B-10 vs IR77298-14-1-2-B-13 : panicle |  |  |  |  |
|  | 1* | 2 | 5,943,967 | 8,879,440 |
|  | 2 | 8 | 2,427,443 | 10,323,025 |
|  | 3 | 11 | 2,957,362 | 4,439,066 |
|  | 4 | 11 | 16,223,493 | 18,664,509 |
|  | 5 | 11 | 19,158,522 | 20,157,500 |
| IR77298-14-1-2-B-10 vs IR77298-14-1-2-B-13 : leaf |  |  |  |  |
|  | 1* | 2 | 5,451,819 | 8,879,440 |
|  | 2 | 2 | 20,215,156 | 21,708,358 |
|  | 3 | 8 | 2,427,443 | 9,824,643 |
|  | 4 | 8 | 17,624,162 | 19,116,801 |
|  | 5 | 11 | 2,458,158 | 4,439,066 |
|  | 6 | 11 | 15,725,381 | 18,189,218 |
|  | 7 | 11 | 19,158,522 | 20,644,445 |
| IR77298-14-1-2-B-10 vs IR77298-14-1-2-B-13 : root |  |  |  |  |
|  | 1 | 5 | 5,951,246 | 6,936,694 |
|  | 2 | 5 | 7,930,186 | 9,428,102 |
|  | 3 | 8 | 4,875,653 | 5,871,741 |

* these aggregation regions overlap with the QTLs mapped in this study
