# Supplementary material for: Genetic, Physiological, and Gene Expression Analyses Reveal That Multiple QTL Enhance Yield of Rice Mega-Variety IR64 under Drought
Source: PLoS One. 2013 May 8;8(5):e62795. doi: 10.1371/journal.pone.0062795 (PMC3648568; doi:10.1371/journal.pone.0062795)
Supplement: Table S5 — Enriched Gramene QTL categories of the subset candidate genes in IR77298-14-1-2-B-10 (+QTL) and IR77298-14-1-2-B-13 (−QTL). (DOCX) [file pone.0062795.s008.docx]

**Table S5.**

| Gramene QTL category | Count of DEGs enriched | Enrichment p value | Gene ID (MSU annotation) |
| --- | --- | --- | --- |
| Abiotic stress\|deep root dry weight\|AQAL006\|c-9 | 51 | 0.0000 | OS09G24580; OS09G24640; OS09G24650; OS09G24660; OS09G24670; OS09G24800; OS09G24910; OS09G24954; OS09G24980; OS09G25050; OS09G25150; OS09G25190; OS09G25310; OS09G25314; OS09G25370; OS09G25410; OS09G25420; OS09G25460; OS09G25470; OS09G25550; OS09G25610; OS09G25620; OS09G25720; OS09G25740; OS09G25760; OS09G25784; OS09G25880; OS09G25910; OS09G25934; OS09G25950; OS09G25970; OS09G26004; OS09G26180; OS09G26190; OS09G26260; OS09G26300; OS09G26310; OS09G26320; OS09G26340; OS09G26350; OS09G26400; OS09G26430; OS09G26440; OS09G26500; OS09G26520; OS09G26530; OS09G26540; OS09G26550; OS09G26554; OS09G26560; OS09G26960 |
| Abiotic stress\|deep root dry weight\|AQAL015\|c-2 | 13 | 0.0000 | OS02G12900; OS02G12939; OS02G13020; OS02G13060; OS02G13090; OS02G13100; OS02G13140; OS02G13160; OS02G13170; OS02G13220; OS02G13304; OS02G13450; OS02G13570 |
| Abiotic stress\|deep root dry weight\|AQAL017\|c-4 | 5 | 0.0113 | OS04G12620; OS04G12678; OS04G12690; OS04G12960; OS04G12970 |
| Abiotic stress\|penetrated root thickness\|AQGC034\|c-10 | 14 | 0.0000 | OS10G35090; OS10G35230; OS10G35240; OS10G35260; OS10G35294; OS10G35436; OS10G35440; OS10G35500; OS10G35520; OS10G35530; OS10G35560; OS10G35630; OS10G35650; OS10G35690 |
| Abiotic stress\|root penetration index\|AQGC016\|c-10 | 14 | 0.0000 | OS10G35090; OS10G35230; OS10G35240; OS10G35260; OS10G35294; OS10G35436; OS10G35440; OS10G35500; OS10G35520; OS10G35530; OS10G35560; OS10G35630; OS10G35650; OS10G35690 |
| Abiotic stress\|root weight\|AQHE052\|c-9 | 32 | 0.0000 | OS09G25550; OS09G25610; OS09G25620; OS09G25720; OS09G25740; OS09G25760; OS09G25784; OS09G25880; OS09G25910; OS09G25934; OS09G25950; OS09G25970; OS09G26004; OS09G26180; OS09G26190; OS09G26260; OS09G26300; OS09G26310; OS09G26320; OS09G26340; OS09G26350; OS09G26400; OS09G26430; OS09G26440; OS09G26500; OS09G26520; OS09G26530; OS09G26540; OS09G26550; OS09G26554; OS09G26560; OS09G26960 |
| Abiotic stress\|root weight\|AQHE055\|c-9 | 32 | 0.0000 | OS09G25550; OS09G25610; OS09G25620; OS09G25720; OS09G25740; OS09G25760; OS09G25784; OS09G25880; OS09G25910; OS09G25934; OS09G25950; OS09G25970; OS09G26004; OS09G26180; OS09G26190; OS09G26260; OS09G26300; OS09G26310; OS09G26320; OS09G26340; OS09G26350; OS09G26400; OS09G26430; OS09G26440; OS09G26500; OS09G26520; OS09G26530; OS09G26540; OS09G26550; OS09G26554; OS09G26560; OS09G26960 |
| Abiotic stress\|root weight\|AQHE059\|c-9 | 32 | 0.0000 | OS09G25550; OS09G25610; OS09G25620; OS09G25720; OS09G25740; OS09G25760; OS09G25784; OS09G25880; OS09G25910; OS09G25934; OS09G25950; OS09G25970; OS09G26004; OS09G26180; OS09G26190; OS09G26260; OS09G26300; OS09G26310; OS09G26320; OS09G26340; OS09G26350; OS09G26400; OS09G26430; OS09G26440; OS09G26500; OS09G26520; OS09G26530; OS09G26540; OS09G26550; OS09G26554; OS09G26560; OS09G26960 |
| Abiotic stress\|root weight\|AQHE085\|c-9 | 32 | 0.0000 | OS09G25550; OS09G25610; OS09G25620; OS09G25720; OS09G25740; OS09G25760; OS09G25784; OS09G25880; OS09G25910; OS09G25934; OS09G25950; OS09G25970; OS09G26004; OS09G26180; OS09G26190; OS09G26260; OS09G26300; OS09G26310; OS09G26320; OS09G26340; OS09G26350; OS09G26400; OS09G26430; OS09G26440; OS09G26500; OS09G26520; OS09G26530; OS09G26540; OS09G26550; OS09G26554; OS09G26560; OS09G26960 |
| Abiotic stress\|root weight\|CQQ39\|c-9 | 27 | 0.0000 | OS09G24580; OS09G24640; OS09G24650; OS09G24660; OS09G24670; OS09G24800; OS09G24910; OS09G24954; OS09G24980; OS09G25050; OS09G25150; OS09G25190; OS09G25310; OS09G25314; OS09G25370; OS09G25410; OS09G25420; OS09G25460; OS09G25470; OS09G25550; OS09G25610; OS09G25620; OS09G25720; OS09G25740; OS09G25760; OS09G25784; OS09G25880 |
| Abiotic stress\|root weight\|CQQ8\|c-9 | 27 | 0.0000 | OS09G24580; OS09G24640; OS09G24650; OS09G24660; OS09G24670; OS09G24800; OS09G24910; OS09G24954; OS09G24980; OS09G25050; OS09G25150; OS09G25190; OS09G25310; OS09G25314; OS09G25370; OS09G25410; OS09G25420; OS09G25460; OS09G25470; OS09G25550; OS09G25610; OS09G25620; OS09G25720; OS09G25740; OS09G25760; OS09G25784; OS09G25880 |
| Anatomy\|leaf area\|AQAI004\|c-9 | 50 | 0.0000 | OS09G24640; OS09G24650; OS09G24660; OS09G24670; OS09G24800; OS09G24910; OS09G24954; OS09G24980; OS09G25050; OS09G25150; OS09G25190; OS09G25310; OS09G25314; OS09G25370; OS09G25410; OS09G25420; OS09G25460; OS09G25470; OS09G25550; OS09G25610; OS09G25620; OS09G25720; OS09G25740; OS09G25760; OS09G25784; OS09G25880; OS09G25910; OS09G25934; OS09G25950; OS09G25970; OS09G26004; OS09G26180; OS09G26190; OS09G26260; OS09G26300; OS09G26310; OS09G26320; OS09G26340; OS09G26350; OS09G26400; OS09G26430; OS09G26440; OS09G26500; OS09G26520; OS09G26530; OS09G26540; OS09G26550; OS09G26554; OS09G26560; OS09G26960 |
| Anatomy\|leaf area\|AQAI014\|c-10 | 27 | 0.0000 | OS10G35090; OS10G35230; OS10G35240; OS10G35260; OS10G35294; OS10G35436; OS10G35440; OS10G35500; OS10G35520; OS10G35530; OS10G35560; OS10G35630; OS10G35650; OS10G35690; OS10G35720; OS10G35730; OS10G35800; OS10G35820; OS10G35990; OS10G36000; OS10G36070; OS10G36090; OS10G36100; OS10G36170; OS10G36210; OS10G36250; OS10G36270 |
| Anatomy\|leaf width\|AQCU222\|c-2 | 4 | 0.0018 | OS02G12900; OS02G12939; OS02G13020; OS02G13060 |
| Anatomy\|panicle length\|AQAB003\|c-10 | 27 | 0.0000 | OS10G35090; OS10G35230; OS10G35240; OS10G35260; OS10G35294; OS10G35436; OS10G35440; OS10G35500; OS10G35520; OS10G35530; OS10G35560; OS10G35630; OS10G35650; OS10G35690; OS10G35720; OS10G35730; OS10G35800; OS10G35820; OS10G35990; OS10G36000; OS10G36070; OS10G36090; OS10G36100; OS10G36170; OS10G36210; OS10G36250; OS10G36270 |
| Anatomy\|panicle length\|AQCU037\|c-10 | 27 | 0.0000 | OS10G35090; OS10G35230; OS10G35240; OS10G35260; OS10G35294; OS10G35436; OS10G35440; OS10G35500; OS10G35520; OS10G35530; OS10G35560; OS10G35630; OS10G35650; OS10G35690; OS10G35720; OS10G35730; OS10G35800; OS10G35820; OS10G35990; OS10G36000; OS10G36070; OS10G36090; OS10G36100; OS10G36170; OS10G36210; OS10G36250; OS10G36270 |
| Anatomy\|panicle length\|AQCU231\|c-2 | 10 | 0.0003 | OS02G13060; OS02G13090; OS02G13100; OS02G13140; OS02G13160; OS02G13170; OS02G13220; OS02G13304; OS02G13450; OS02G13570 |
| Anatomy\|panicle length\|AQCU232\|c-2 | 4 | 0.0018 | OS02G12900; OS02G12939; OS02G13020; OS02G13060 |
| Anatomy\|panicle length\|AQDY116\|c-9 | 32 | 0.0000 | OS09G25550; OS09G25610; OS09G25620; OS09G25720; OS09G25740; OS09G25760; OS09G25784; OS09G25880; OS09G25910; OS09G25934; OS09G25950; OS09G25970; OS09G26004; OS09G26180; OS09G26190; OS09G26260; OS09G26300; OS09G26310; OS09G26320; OS09G26340; OS09G26350; OS09G26400; OS09G26430; OS09G26440; OS09G26500; OS09G26520; OS09G26530; OS09G26540; OS09G26550; OS09G26554; OS09G26560; OS09G26960 |
| Anatomy\|panicle length\|AQDY117\|c-9 | 32 | 0.0000 | OS09G25550; OS09G25610; OS09G25620; OS09G25720; OS09G25740; OS09G25760; OS09G25784; OS09G25880; OS09G25910; OS09G25934; OS09G25950; OS09G25970; OS09G26004; OS09G26180; OS09G26190; OS09G26260; OS09G26300; OS09G26310; OS09G26320; OS09G26340; OS09G26350; OS09G26400; OS09G26430; OS09G26440; OS09G26500; OS09G26520; OS09G26530; OS09G26540; OS09G26550; OS09G26554; OS09G26560; OS09G26960 |
| Anatomy\|panicle length\|AQDY126\|c-10 | 27 | 0.0000 | OS10G35090; OS10G35230; OS10G35240; OS10G35260; OS10G35294; OS10G35436; OS10G35440; OS10G35500; OS10G35520; OS10G35530; OS10G35560; OS10G35630; OS10G35650; OS10G35690; OS10G35720; OS10G35730; OS10G35800; OS10G35820; OS10G35990; OS10G36000; OS10G36070; OS10G36090; OS10G36100; OS10G36170; OS10G36210; OS10G36250; OS10G36270 |
| Anatomy\|panicle length\|AQDY127\|c-10 | 27 | 0.0000 | OS10G35090; OS10G35230; OS10G35240; OS10G35260; OS10G35294; OS10G35436; OS10G35440; OS10G35500; OS10G35520; OS10G35530; OS10G35560; OS10G35630; OS10G35650; OS10G35690; OS10G35720; OS10G35730; OS10G35800; OS10G35820; OS10G35990; OS10G36000; OS10G36070; OS10G36090; OS10G36100; OS10G36170; OS10G36210; OS10G36250; OS10G36270 |
| Anatomy\|panicle length\|AQFJ049\|c-9 | 46 | 0.0000 | OS09G24800; OS09G24910; OS09G24954; OS09G24980; OS09G25050; OS09G25150; OS09G25190; OS09G25310; OS09G25314; OS09G25370; OS09G25410; OS09G25420; OS09G25460; OS09G25470; OS09G25550; OS09G25610; OS09G25620; OS09G25720; OS09G25740; OS09G25760; OS09G25784; OS09G25880; OS09G25910; OS09G25934; OS09G25950; OS09G25970; OS09G26004; OS09G26180; OS09G26190; OS09G26260; OS09G26300; OS09G26310; OS09G26320; OS09G26340; OS09G26350; OS09G26400; OS09G26430; OS09G26440; OS09G26500; OS09G26520; OS09G26530; OS09G26540; OS09G26550; OS09G26554; OS09G26560; OS09G26960 |
| Anatomy\|panicle length\|AQFW101\|c-2 | 10 | 0.0003 | OS02G13060; OS02G13090; OS02G13100; OS02G13140; OS02G13160; OS02G13170; OS02G13220; OS02G13304; OS02G13450; OS02G13570 |
| Anatomy\|panicle length\|AQGM012\|c-10 | 27 | 0.0000 | OS10G35090; OS10G35230; OS10G35240; OS10G35260; OS10G35294; OS10G35436; OS10G35440; OS10G35500; OS10G35520; OS10G35530; OS10G35560; OS10G35630; OS10G35650; OS10G35690; OS10G35720; OS10G35730; OS10G35800; OS10G35820; OS10G35990; OS10G36000; OS10G36070; OS10G36090; OS10G36100; OS10G36170; OS10G36210; OS10G36250; OS10G36270 |
| Anatomy\|panicle length\|CQAS108\|c-9 | 24 | 0.0000 | OS09G25910; OS09G25934; OS09G25950; OS09G25970; OS09G26004; OS09G26180; OS09G26190; OS09G26260; OS09G26300; OS09G26310; OS09G26320; OS09G26340; OS09G26350; OS09G26400; OS09G26430; OS09G26440; OS09G26500; OS09G26520; OS09G26530; OS09G26540; OS09G26550; OS09G26554; OS09G26560; OS09G26960 |
| Anatomy\|panicle length\|CQAS121\|c-10 | 18 | 0.0000 | OS10G35090; OS10G35230; OS10G35240; OS10G35260; OS10G35294; OS10G35436; OS10G35440; OS10G35500; OS10G35520; OS10G35530; OS10G35560; OS10G35630; OS10G35650; OS10G35690; OS10G35720; OS10G35730; OS10G35800; OS10G35820 |
| Anatomy\|root length\|AQA020\|c-9 | 32 | 0.0000 | OS09G24580; OS09G24640; OS09G24650; OS09G24660; OS09G24670; OS09G24800; OS09G24910; OS09G24954; OS09G24980; OS09G25050; OS09G25150; OS09G25190; OS09G25310; OS09G25314; OS09G25370; OS09G25410; OS09G25420; OS09G25460; OS09G25470; OS09G25550; OS09G25610; OS09G25620; OS09G25720; OS09G25740; OS09G25760; OS09G25784; OS09G25880; OS09G25910; OS09G25934; OS09G25950; OS09G25970; OS09G26004 |
| Anatomy\|root length\|AQHE006\|c-9 | 32 | 0.0000 | OS09G25550; OS09G25610; OS09G25620; OS09G25720; OS09G25740; OS09G25760; OS09G25784; OS09G25880; OS09G25910; OS09G25934; OS09G25950; OS09G25970; OS09G26004; OS09G26180; OS09G26190; OS09G26260; OS09G26300; OS09G26310; OS09G26320; OS09G26340; OS09G26350; OS09G26400; OS09G26430; OS09G26440; OS09G26500; OS09G26520; OS09G26530; OS09G26540; OS09G26550; OS09G26554; OS09G26560; OS09G26960 |
| Anatomy\|root length\|CQAI23\|c-9 | 27 | 0.0000 | OS09G24580; OS09G24640; OS09G24650; OS09G24660; OS09G24670; OS09G24800; OS09G24910; OS09G24954; OS09G24980; OS09G25050; OS09G25150; OS09G25190; OS09G25310; OS09G25314; OS09G25370; OS09G25410; OS09G25420; OS09G25460; OS09G25470; OS09G25550; OS09G25610; OS09G25620; OS09G25720; OS09G25740; OS09G25760; OS09G25784; OS09G25880 |
| Anatomy\|seminal root length\|CQS9\|c-9 | 27 | 0.0000 | OS09G24580; OS09G24640; OS09G24650; OS09G24660; OS09G24670; OS09G24800; OS09G24910; OS09G24954; OS09G24980; OS09G25050; OS09G25150; OS09G25190; OS09G25310; OS09G25314; OS09G25370; OS09G25410; OS09G25420; OS09G25460; OS09G25470; OS09G25550; OS09G25610; OS09G25620; OS09G25720; OS09G25740; OS09G25760; OS09G25784; OS09G25880 |
| Anatomy\|spikelet density\|CQAS124\|c-10 | 18 | 0.0000 | OS10G35090; OS10G35230; OS10G35240; OS10G35260; OS10G35294; OS10G35436; OS10G35440; OS10G35500; OS10G35520; OS10G35530; OS10G35560; OS10G35630; OS10G35650; OS10G35690; OS10G35720; OS10G35730; OS10G35800; OS10G35820 |
| Anatomy\|spikelet density\|CQAS20\|c-2 | 10 | 0.0000 | OS02G13060; OS02G13090; OS02G13100; OS02G13140; OS02G13160; OS02G13170; OS02G13220; OS02G13304; OS02G13450; OS02G13570 |
| Development\|reproductive growth time\|CQA33\|c-10 | 27 | 0.0000 | OS10G35090; OS10G35230; OS10G35240; OS10G35260; OS10G35294; OS10G35436; OS10G35440; OS10G35500; OS10G35520; OS10G35530; OS10G35560; OS10G35630; OS10G35650; OS10G35690; OS10G35720; OS10G35730; OS10G35800; OS10G35820; OS10G35990; OS10G36000; OS10G36070; OS10G36090; OS10G36100; OS10G36170; OS10G36210; OS10G36250; OS10G36270 |
| Development\|reproductive growth time\|CQA43\|c-2 | 13 | 0.0000 | OS02G12900; OS02G12939; OS02G13020; OS02G13060; OS02G13090; OS02G13100; OS02G13140; OS02G13160; OS02G13170; OS02G13220; OS02G13304; OS02G13450; OS02G13570 |
| Development\|shoot elongation rate\|CQE73\|c-9 | 19 | 0.0000 | OS09G24580; OS09G24640; OS09G24650; OS09G24660; OS09G24670; OS09G24800; OS09G24910; OS09G24954; OS09G24980; OS09G25050; OS09G25150; OS09G25190; OS09G25310; OS09G25314; OS09G25370; OS09G25410; OS09G25420; OS09G25460; OS09G25470 |
| Development\|shoot elongation rate\|CQE74\|c-9 | 51 | 0.0000 | OS09G24580; OS09G24640; OS09G24650; OS09G24660; OS09G24670; OS09G24800; OS09G24910; OS09G24954; OS09G24980; OS09G25050; OS09G25150; OS09G25190; OS09G25310; OS09G25314; OS09G25370; OS09G25410; OS09G25420; OS09G25460; OS09G25470; OS09G25550; OS09G25610; OS09G25620; OS09G25720; OS09G25740; OS09G25760; OS09G25784; OS09G25880; OS09G25910; OS09G25934; OS09G25950; OS09G25970; OS09G26004; OS09G26180; OS09G26190; OS09G26260; OS09G26300; OS09G26310; OS09G26320; OS09G26340; OS09G26350; OS09G26400; OS09G26430; OS09G26440; OS09G26500; OS09G26520; OS09G26530; OS09G26540; OS09G26550; OS09G26554; OS09G26560; OS09G26960 |
| Development\|vegetative growth time\|CQA32\|c-10 | 27 | 0.0000 | OS10G35090; OS10G35230; OS10G35240; OS10G35260; OS10G35294; OS10G35436; OS10G35440; OS10G35500; OS10G35520; OS10G35530; OS10G35560; OS10G35630; OS10G35650; OS10G35690; OS10G35720; OS10G35730; OS10G35800; OS10G35820; OS10G35990; OS10G36000; OS10G36070; OS10G36090; OS10G36100; OS10G36170; OS10G36210; OS10G36250; OS10G36270 |
| Development\|vegetative growth time\|CQA42\|c-2 | 13 | 0.0000 | OS02G12900; OS02G12939; OS02G13020; OS02G13060; OS02G13090; OS02G13100; OS02G13140; OS02G13160; OS02G13170; OS02G13220; OS02G13304; OS02G13450; OS02G13570 |
| Sterility or fertility\|pollen fertility\|AQBI006\|c-10 | 27 | 0.0000 | OS10G35090; OS10G35230; OS10G35240; OS10G35260; OS10G35294; OS10G35436; OS10G35440; OS10G35500; OS10G35520; OS10G35530; OS10G35560; OS10G35630; OS10G35650; OS10G35690; OS10G35720; OS10G35730; OS10G35800; OS10G35820; OS10G35990; OS10G36000; OS10G36070; OS10G36090; OS10G36100; OS10G36170; OS10G36210; OS10G36250; OS10G36270 |
| Sterility or fertility\|spikelet fertility\|AQAK023\|c-10 | 20 | 0.0000 | OS10G35090; OS10G35230; OS10G35240; OS10G35260; OS10G35294; OS10G35436; OS10G35440; OS10G35500; OS10G35520; OS10G35530; OS10G35560; OS10G35630; OS10G35650; OS10G35690; OS10G35720; OS10G35730; OS10G35800; OS10G35820; OS10G35990; OS10G36000 |
| Sterility or fertility\|spikelet fertility\|AQDQ020\|c-2 | 13 | 0.0000 | OS02G12900; OS02G12939; OS02G13020; OS02G13060; OS02G13090; OS02G13100; OS02G13140; OS02G13160; OS02G13170; OS02G13220; OS02G13304; OS02G13450; OS02G13570 |
| Sterility or fertility\|spikelet fertility\|AQFW015\|c-2 | 10 | 0.0003 | OS02G13060; OS02G13090; OS02G13100; OS02G13140; OS02G13160; OS02G13170; OS02G13220; OS02G13304; OS02G13450; OS02G13570 |
| Sterility or fertility\|spikelet fertility\|AQFW259\|c-2 | 4 | 0.0018 | OS02G12900; OS02G12939; OS02G13020; OS02G13060 |
| Vigor\|root dry weight\|AQEX024\|c-4 | 5 | 0.0033 | OS04G12620; OS04G12678; OS04G12690; OS04G12960; OS04G12970 |
| Vigor\|root dry weight\|AQGI088\|c-9 | 50 | 0.0000 | OS09G24640; OS09G24650; OS09G24660; OS09G24670; OS09G24800; OS09G24910; OS09G24954; OS09G24980; OS09G25050; OS09G25150; OS09G25190; OS09G25310; OS09G25314; OS09G25370; OS09G25410; OS09G25420; OS09G25460; OS09G25470; OS09G25550; OS09G25610; OS09G25620; OS09G25720; OS09G25740; OS09G25760; OS09G25784; OS09G25880; OS09G25910; OS09G25934; OS09G25950; OS09G25970; OS09G26004; OS09G26180; OS09G26190; OS09G26260; OS09G26300; OS09G26310; OS09G26320; OS09G26340; OS09G26350; OS09G26400; OS09G26430; OS09G26440; OS09G26500; OS09G26520; OS09G26530; OS09G26540; OS09G26550; OS09G26554; OS09G26560; OS09G26960 |
| Vigor\|root dry weight\|AQGI097\|c-9 | 50 | 0.0000 | OS09G24640; OS09G24650; OS09G24660; OS09G24670; OS09G24800; OS09G24910; OS09G24954; OS09G24980; OS09G25050; OS09G25150; OS09G25190; OS09G25310; OS09G25314; OS09G25370; OS09G25410; OS09G25420; OS09G25460; OS09G25470; OS09G25550; OS09G25610; OS09G25620; OS09G25720; OS09G25740; OS09G25760; OS09G25784; OS09G25880; OS09G25910; OS09G25934; OS09G25950; OS09G25970; OS09G26004; OS09G26180; OS09G26190; OS09G26260; OS09G26300; OS09G26310; OS09G26320; OS09G26340; OS09G26350; OS09G26400; OS09G26430; OS09G26440; OS09G26500; OS09G26520; OS09G26530; OS09G26540; OS09G26550; OS09G26554; OS09G26560; OS09G26960 |
| Vigor\|seedling vigor\|AQEP006\|c-9 | 51 | 0.0000 | OS09G24580; OS09G24640; OS09G24650; OS09G24660; OS09G24670; OS09G24800; OS09G24910; OS09G24954; OS09G24980; OS09G25050; OS09G25150; OS09G25190; OS09G25310; OS09G25314; OS09G25370; OS09G25410; OS09G25420; OS09G25460; OS09G25470; OS09G25550; OS09G25610; OS09G25620; OS09G25720; OS09G25740; OS09G25760; OS09G25784; OS09G25880; OS09G25910; OS09G25934; OS09G25950; OS09G25970; OS09G26004; OS09G26180; OS09G26190; OS09G26260; OS09G26300; OS09G26310; OS09G26320; OS09G26340; OS09G26350; OS09G26400; OS09G26430; OS09G26440; OS09G26500; OS09G26520; OS09G26530; OS09G26540; OS09G26550; OS09G26554; OS09G26560; OS09G26960 |
| Vigor\|tiller number\|AQA015\|c-9 | 32 | 0.0000 | OS09G24580; OS09G24640; OS09G24650; OS09G24660; OS09G24670; OS09G24800; OS09G24910; OS09G24954; OS09G24980; OS09G25050; OS09G25150; OS09G25190; OS09G25310; OS09G25314; OS09G25370; OS09G25410; OS09G25420; OS09G25460; OS09G25470; OS09G25550; OS09G25610; OS09G25620; OS09G25720; OS09G25740; OS09G25760; OS09G25784; OS09G25880; OS09G25910; OS09G25934; OS09G25950; OS09G25970; OS09G26004 |
| Vigor\|tiller number\|AQDY118\|c-9 | 32 | 0.0000 | OS09G25550; OS09G25610; OS09G25620; OS09G25720; OS09G25740; OS09G25760; OS09G25784; OS09G25880; OS09G25910; OS09G25934; OS09G25950; OS09G25970; OS09G26004; OS09G26180; OS09G26190; OS09G26260; OS09G26300; OS09G26310; OS09G26320; OS09G26340; OS09G26350; OS09G26400; OS09G26430; OS09G26440; OS09G26500; OS09G26520; OS09G26530; OS09G26540; OS09G26550; OS09G26554; OS09G26560; OS09G26960 |
| Vigor\|tiller number\|AQDY137\|c-10 | 27 | 0.0000 | OS10G35090; OS10G35230; OS10G35240; OS10G35260; OS10G35294; OS10G35436; OS10G35440; OS10G35500; OS10G35520; OS10G35530; OS10G35560; OS10G35630; OS10G35650; OS10G35690; OS10G35720; OS10G35730; OS10G35800; OS10G35820; OS10G35990; OS10G36000; OS10G36070; OS10G36090; OS10G36100; OS10G36170; OS10G36210; OS10G36250; OS10G36270 |
| Vigor\|tiller number\|AQHR023\|c-9b | 50 | 0.0000 | OS09G24640; OS09G24650; OS09G24660; OS09G24670; OS09G24800; OS09G24910; OS09G24954; OS09G24980; OS09G25050; OS09G25150; OS09G25190; OS09G25310; OS09G25314; OS09G25370; OS09G25410; OS09G25420; OS09G25460; OS09G25470; OS09G25550; OS09G25610; OS09G25620; OS09G25720; OS09G25740; OS09G25760; OS09G25784; OS09G25880; OS09G25910; OS09G25934; OS09G25950; OS09G25970; OS09G26004; OS09G26180; OS09G26190; OS09G26260; OS09G26300; OS09G26310; OS09G26320; OS09G26340; OS09G26350; OS09G26400; OS09G26430; OS09G26440; OS09G26500; OS09G26520; OS09G26530; OS09G26540; OS09G26550; OS09G26554; OS09G26560; OS09G26960 |
| Vigor\|tiller number\|CQAI18\|c-9 | 27 | 0.0000 | OS09G24580; OS09G24640; OS09G24650; OS09G24660; OS09G24670; OS09G24800; OS09G24910; OS09G24954; OS09G24980; OS09G25050; OS09G25150; OS09G25190; OS09G25310; OS09G25314; OS09G25370; OS09G25410; OS09G25420; OS09G25460; OS09G25470; OS09G25550; OS09G25610; OS09G25620; OS09G25720; OS09G25740; OS09G25760; OS09G25784; OS09G25880 |
| Yield\|1000-seed weight\|AQCF013\|c-10 | 9 | 0.0000 | OS10G35990; OS10G36000; OS10G36070; OS10G36090; OS10G36100; OS10G36170; OS10G36210; OS10G36250; OS10G36270 |
| Yield\|1000-seed weight\|AQCF024\|c-10 | 9 | 0.0000 | OS10G35990; OS10G36000; OS10G36070; OS10G36090; OS10G36100; OS10G36170; OS10G36210; OS10G36250; OS10G36270 |
| Yield\|1000-seed weight\|AQCF045\|c-10 | 9 | 0.0000 | OS10G35990; OS10G36000; OS10G36070; OS10G36090; OS10G36100; OS10G36170; OS10G36210; OS10G36250; OS10G36270 |
| Yield\|1000-seed weight\|AQDR008\|c-10 | 27 | 0.0000 | OS10G35090; OS10G35230; OS10G35240; OS10G35260; OS10G35294; OS10G35436; OS10G35440; OS10G35500; OS10G35520; OS10G35530; OS10G35560; OS10G35630; OS10G35650; OS10G35690; OS10G35720; OS10G35730; OS10G35800; OS10G35820; OS10G35990; OS10G36000; OS10G36070; OS10G36090; OS10G36100; OS10G36170; OS10G36210; OS10G36250; OS10G36270 |
| Yield\|grain number\|AQDR015\|c-10 | 27 | 0.0000 | OS10G35090; OS10G35230; OS10G35240; OS10G35260; OS10G35294; OS10G35436; OS10G35440; OS10G35500; OS10G35520; OS10G35530; OS10G35560; OS10G35630; OS10G35650; OS10G35690; OS10G35720; OS10G35730; OS10G35800; OS10G35820; OS10G35990; OS10G36000; OS10G36070; OS10G36090; OS10G36100; OS10G36170; OS10G36210; OS10G36250; OS10G36270 |
| Yield\|grain number\|AQDR049\|c-2 | 13 | 0.0000 | OS02G12900; OS02G12939; OS02G13020; OS02G13060; OS02G13090; OS02G13100; OS02G13140; OS02G13160; OS02G13170; OS02G13220; OS02G13304; OS02G13450; OS02G13570 |
| Yield\|grain yield per plant\|AQCN013\|c-9 | 27 | 0.0000 | OS09G24580; OS09G24640; OS09G24650; OS09G24660; OS09G24670; OS09G24800; OS09G24910; OS09G24954; OS09G24980; OS09G25050; OS09G25150; OS09G25190; OS09G25310; OS09G25314; OS09G25370; OS09G25410; OS09G25420; OS09G25460; OS09G25470; OS09G25550; OS09G25610; OS09G25620; OS09G25720; OS09G25740; OS09G25760; OS09G25784; OS09G25880 |
| Yield\|grain yield\|AQF081\|c-9 | 19 | 0.0000 | OS09G24580; OS09G24640; OS09G24650; OS09G24660; OS09G24670; OS09G24800; OS09G24910; OS09G24954; OS09G24980; OS09G25050; OS09G25150; OS09G25190; OS09G25310; OS09G25314; OS09G25370; OS09G25410; OS09G25420; OS09G25460; OS09G25470 |
| Yield\|panicle number\|AQCQ008\|c-10 | 27 | 0.0000 | OS10G35090; OS10G35230; OS10G35240; OS10G35260; OS10G35294; OS10G35436; OS10G35440; OS10G35500; OS10G35520; OS10G35530; OS10G35560; OS10G35630; OS10G35650; OS10G35690; OS10G35720; OS10G35730; OS10G35800; OS10G35820; OS10G35990; OS10G36000; OS10G36070; OS10G36090; OS10G36100; OS10G36170; OS10G36210; OS10G36250; OS10G36270 |
| Yield\|panicle number\|AQDY130\|c-10 | 27 | 0.0000 | OS10G35090; OS10G35230; OS10G35240; OS10G35260; OS10G35294; OS10G35436; OS10G35440; OS10G35500; OS10G35520; OS10G35530; OS10G35560; OS10G35630; OS10G35650; OS10G35690; OS10G35720; OS10G35730; OS10G35800; OS10G35820; OS10G35990; OS10G36000; OS10G36070; OS10G36090; OS10G36100; OS10G36170; OS10G36210; OS10G36250; OS10G36270 |
| Yield\|panicle number\|AQDY131\|c-10 | 27 | 0.0000 | OS10G35090; OS10G35230; OS10G35240; OS10G35260; OS10G35294; OS10G35436; OS10G35440; OS10G35500; OS10G35520; OS10G35530; OS10G35560; OS10G35630; OS10G35650; OS10G35690; OS10G35720; OS10G35730; OS10G35800; OS10G35820; OS10G35990; OS10G36000; OS10G36070; OS10G36090; OS10G36100; OS10G36170; OS10G36210; OS10G36250; OS10G36270 |
| Yield\|panicle number\|AQDY132\|c-10 | 27 | 0.0000 | OS10G35090; OS10G35230; OS10G35240; OS10G35260; OS10G35294; OS10G35436; OS10G35440; OS10G35500; OS10G35520; OS10G35530; OS10G35560; OS10G35630; OS10G35650; OS10G35690; OS10G35720; OS10G35730; OS10G35800; OS10G35820; OS10G35990; OS10G36000; OS10G36070; OS10G36090; OS10G36100; OS10G36170; OS10G36210; OS10G36250; OS10G36270 |
| Yield\|panicle number\|AQDY133\|c-10 | 27 | 0.0000 | OS10G35090; OS10G35230; OS10G35240; OS10G35260; OS10G35294; OS10G35436; OS10G35440; OS10G35500; OS10G35520; OS10G35530; OS10G35560; OS10G35630; OS10G35650; OS10G35690; OS10G35720; OS10G35730; OS10G35800; OS10G35820; OS10G35990; OS10G36000; OS10G36070; OS10G36090; OS10G36100; OS10G36170; OS10G36210; OS10G36250; OS10G36270 |
| Yield\|panicle number\|AQDY135\|c-10 | 27 | 0.0000 | OS10G35090; OS10G35230; OS10G35240; OS10G35260; OS10G35294; OS10G35436; OS10G35440; OS10G35500; OS10G35520; OS10G35530; OS10G35560; OS10G35630; OS10G35650; OS10G35690; OS10G35720; OS10G35730; OS10G35800; OS10G35820; OS10G35990; OS10G36000; OS10G36070; OS10G36090; OS10G36100; OS10G36170; OS10G36210; OS10G36250; OS10G36270 |
| Yield\|panicle number\|AQFF065\|c-4 | 5 | 0.0073 | OS04G12620; OS04G12678; OS04G12690; OS04G12960; OS04G12970 |
| Yield\|panicle number\|AQFJ010\|c-9 | 46 | 0.0000 | OS09G24800; OS09G24910; OS09G24954; OS09G24980; OS09G25050; OS09G25150; OS09G25190; OS09G25310; OS09G25314; OS09G25370; OS09G25410; OS09G25420; OS09G25460; OS09G25470; OS09G25550; OS09G25610; OS09G25620; OS09G25720; OS09G25740; OS09G25760; OS09G25784; OS09G25880; OS09G25910; OS09G25934; OS09G25950; OS09G25970; OS09G26004; OS09G26180; OS09G26190; OS09G26260; OS09G26300; OS09G26310; OS09G26320; OS09G26340; OS09G26350; OS09G26400; OS09G26430; OS09G26440; OS09G26500; OS09G26520; OS09G26530; OS09G26540; OS09G26550; OS09G26554; OS09G26560; OS09G26960 |
| Yield\|panicle number\|CQAS122\|c-10 | 18 | 0.0000 | OS10G35090; OS10G35230; OS10G35240; OS10G35260; OS10G35294; OS10G35436; OS10G35440; OS10G35500; OS10G35520; OS10G35530; OS10G35560; OS10G35630; OS10G35650; OS10G35690; OS10G35720; OS10G35730; OS10G35800; OS10G35820 |
| Yield\|seed number\|AQBK041\|c-2 | 13 | 0.0010 | OS02G12900; OS02G12939; OS02G13020; OS02G13060; OS02G13090; OS02G13100; OS02G13140; OS02G13160; OS02G13170; OS02G13220; OS02G13304; OS02G13450; OS02G13570 |
| Yield\|seed number\|AQBK043\|c-10 | 27 | 0.0000 | OS10G35090; OS10G35230; OS10G35240; OS10G35260; OS10G35294; OS10G35436; OS10G35440; OS10G35500; OS10G35520; OS10G35530; OS10G35560; OS10G35630; OS10G35650; OS10G35690; OS10G35720; OS10G35730; OS10G35800; OS10G35820; OS10G35990; OS10G36000; OS10G36070; OS10G36090; OS10G36100; OS10G36170; OS10G36210; OS10G36250; OS10G36270 |
| Yield\|seed number\|CQAS129\|c-10 | 20 | 0.0000 | OS10G35090; OS10G35230; OS10G35240; OS10G35260; OS10G35294; OS10G35436; OS10G35440; OS10G35500; OS10G35520; OS10G35530; OS10G35560; OS10G35630; OS10G35650; OS10G35690; OS10G35720; OS10G35730; OS10G35800; OS10G35820; OS10G35990; OS10G36000 |
| Yield\|seed set percent\|AQBK051\|c-10 | 27 | 0.0000 | OS10G35090; OS10G35230; OS10G35240; OS10G35260; OS10G35294; OS10G35436; OS10G35440; OS10G35500; OS10G35520; OS10G35530; OS10G35560; OS10G35630; OS10G35650; OS10G35690; OS10G35720; OS10G35730; OS10G35800; OS10G35820; OS10G35990; OS10G36000; OS10G36070; OS10G36090; OS10G36100; OS10G36170; OS10G36210; OS10G36250; OS10G36270 |
| Yield\|seed set percent\|AQBK055\|c-10 | 27 | 0.0000 | OS10G35090; OS10G35230; OS10G35240; OS10G35260; OS10G35294; OS10G35436; OS10G35440; OS10G35500; OS10G35520; OS10G35530; OS10G35560; OS10G35630; OS10G35650; OS10G35690; OS10G35720; OS10G35730; OS10G35800; OS10G35820; OS10G35990; OS10G36000; OS10G36070; OS10G36090; OS10G36100; OS10G36170; OS10G36210; OS10G36250; OS10G36270 |
| Yield\|seed set percent\|AQFE065\|c-10 | 27 | 0.0000 | OS10G35090; OS10G35230; OS10G35240; OS10G35260; OS10G35294; OS10G35436; OS10G35440; OS10G35500; OS10G35520; OS10G35530; OS10G35560; OS10G35630; OS10G35650; OS10G35690; OS10G35720; OS10G35730; OS10G35800; OS10G35820; OS10G35990; OS10G36000; OS10G36070; OS10G36090; OS10G36100; OS10G36170; OS10G36210; OS10G36250; OS10G36270 |
| Yield\|seed set percent\|AQFE066\|c-10 | 27 | 0.0000 | OS10G35090; OS10G35230; OS10G35240; OS10G35260; OS10G35294; OS10G35436; OS10G35440; OS10G35500; OS10G35520; OS10G35530; OS10G35560; OS10G35630; OS10G35650; OS10G35690; OS10G35720; OS10G35730; OS10G35800; OS10G35820; OS10G35990; OS10G36000; OS10G36070; OS10G36090; OS10G36100; OS10G36170; OS10G36210; OS10G36250; OS10G36270 |
| Yield\|seed set percent\|CQAS126\|c-10 | 20 | 0.0000 | OS10G35090; OS10G35230; OS10G35240; OS10G35260; OS10G35294; OS10G35436; OS10G35440; OS10G35500; OS10G35520; OS10G35530; OS10G35560; OS10G35630; OS10G35650; OS10G35690; OS10G35720; OS10G35730; OS10G35800; OS10G35820; OS10G35990; OS10G36000 |
| Yield\|spikelet number\|AQBK031\|c-2 | 13 | 0.0003 | OS02G12900; OS02G12939; OS02G13020; OS02G13060; OS02G13090; OS02G13100; OS02G13140; OS02G13160; OS02G13170; OS02G13220; OS02G13304; OS02G13450; OS02G13570 |
| Yield\|spikelet number\|AQBK037\|c-9 | 51 | 0.0000 | OS09G24580; OS09G24640; OS09G24650; OS09G24660; OS09G24670; OS09G24800; OS09G24910; OS09G24954; OS09G24980; OS09G25050; OS09G25150; OS09G25190; OS09G25310; OS09G25314; OS09G25370; OS09G25410; OS09G25420; OS09G25460; OS09G25470; OS09G25550; OS09G25610; OS09G25620; OS09G25720; OS09G25740; OS09G25760; OS09G25784; OS09G25880; OS09G25910; OS09G25934; OS09G25950; OS09G25970; OS09G26004; OS09G26180; OS09G26190; OS09G26260; OS09G26300; OS09G26310; OS09G26320; OS09G26340; OS09G26350; OS09G26400; OS09G26430; OS09G26440; OS09G26500; OS09G26520; OS09G26530; OS09G26540; OS09G26550; OS09G26554; OS09G26560; OS09G26960 |
| Yield\|spikelet number\|AQDQ019\|c-2 | 13 | 0.0000 | OS02G12900; OS02G12939; OS02G13020; OS02G13060; OS02G13090; OS02G13100; OS02G13140; OS02G13160; OS02G13170; OS02G13220; OS02G13304; OS02G13450; OS02G13570 |
| Yield\|spikelet number\|CQAS110\|c-9 | 32 | 0.0000 | OS09G25550; OS09G25610; OS09G25620; OS09G25720; OS09G25740; OS09G25760; OS09G25784; OS09G25880; OS09G25910; OS09G25934; OS09G25950; OS09G25970; OS09G26004; OS09G26180; OS09G26190; OS09G26260; OS09G26300; OS09G26310; OS09G26320; OS09G26340; OS09G26350; OS09G26400; OS09G26430; OS09G26440; OS09G26500; OS09G26520; OS09G26530; OS09G26540; OS09G26550; OS09G26554; OS09G26560; OS09G26960 |
| Yield\|spikelet number\|CQAS123\|c-10 | 18 | 0.0000 | OS10G35090; OS10G35230; OS10G35240; OS10G35260; OS10G35294; OS10G35436; OS10G35440; OS10G35500; OS10G35520; OS10G35530; OS10G35560; OS10G35630; OS10G35650; OS10G35690; OS10G35720; OS10G35730; OS10G35800; OS10G35820 |
| Yield\|spikelet number\|CQN56\|c-2 | 13 | 0.0000 | OS02G12900; OS02G12939; OS02G13020; OS02G13060; OS02G13090; OS02G13100; OS02G13140; OS02G13160; OS02G13170; OS02G13220; OS02G13304; OS02G13450; OS02G13570 |
| Yield\|total biomass yield\|AQGI246\|c-10 | 26 | 0.0000 | OS10G35230; OS10G35240; OS10G35260; OS10G35294; OS10G35436; OS10G35440; OS10G35500; OS10G35520; OS10G35530; OS10G35560; OS10G35630; OS10G35650; OS10G35690; OS10G35720; OS10G35730; OS10G35800; OS10G35820; OS10G35990; OS10G36000; OS10G36070; OS10G36090; OS10G36100; OS10G36170; OS10G36210; OS10G36250; OS10G36270 |
| Yield\|total biomass yield\|AQHE109\|c-9 | 32 | 0.0000 | OS09G25550; OS09G25610; OS09G25620; OS09G25720; OS09G25740; OS09G25760; OS09G25784; OS09G25880; OS09G25910; OS09G25934; OS09G25950; OS09G25970; OS09G26004; OS09G26180; OS09G26190; OS09G26260; OS09G26300; OS09G26310; OS09G26320; OS09G26340; OS09G26350; OS09G26400; OS09G26430; OS09G26440; OS09G26500; OS09G26520; OS09G26530; OS09G26540; OS09G26550; OS09G26554; OS09G26560; OS09G26960 |
