# Supplementary material for: Genetic, Physiological, and Gene Expression Analyses Reveal That Multiple QTL Enhance Yield of Rice Mega-Variety IR64 under Drought
Source: PLoS One. 2013 May 8;8(5):e62795. doi: 10.1371/journal.pone.0062795 (PMC3648568; doi:10.1371/journal.pone.0062795)
Supplement: Table S6 — Performance of 84 IR64-NILs under non –stress (NS) and drought stress (S) conditions. (DOCX) [file pone.0062795.s009.docx]

**Table S6.**

| **ENTNO** | **DESIGNATION** | **DTF-NS** | **DTF-S** | **HTAVE_NS** | **HTAVE_S** | **GYKGPHA_NS** | **GYKGPHA_S** |
| --- | --- | --- | --- | --- | --- | --- | --- |
| 1 | IR 87707-342-B-B-B | 79 | 87 | 95 | 81 | 5997.6 | 2684.1 |
| 2 | IR 87706-388-B-B-B | 78 | 86 | 91 | 79 | 6225.2 | 2757.4 |
| 3 | IR 87707-204-B-B-B | 81 | 87 | 91 | 79 | 6997.6 | 3074.0 |
| 4 | IR 87707-446-B-B-B | 80 | 86 | 98 | 81 | 4387.6 | 2999.6 |
| 5 | IR 87707-445-B-B-B | 77 | 87 | 96 | 70 | 5844.3 | 3022.7 |
| 6 | IR 87707-110-B-B-B | 81 | 90 | 97 | 77 | 6006.1 | 2101.8 |
| 7 | IR 87707-118-B-B-B | 81 | 88 | 96 | 81 | 6429.1 | 2682.1 |
| 8 | IR 87707-443-B-B-B | 80 | 85 | 100 | 79 | 5688.8 | 2647.3 |
| 9 | IR 87707-183-B-B-B | 79 | 86 | 98 | 80 | 5656.1 | 2365.1 |
| 10 | IR 87707-515-B-B-B | 79 | 87 | 95 | 76 | 5778.5 | 2871.8 |
| 11 | IR 87707-248-B-B-B | 80 | 87 | 100 | 82 | 6618.2 | 2591.1 |
| 12 | IR 87707-186-B-B-B | 78 | 86 | 99 | 81 | 6103.6 | 2631.6 |
| 13 | IR 87729-27-B-B-B | 82 | 90 | 94 | 71 | 6589.1 | 1516.3 |
| 14 | IR 87707-440-B-B-B | 78 | 86 | 89 | 69 | 5915.8 | 2678.1 |
| 15 | IR 87729-69-B-B-B | 83 | 90 | 91 | 78 | 6308.2 | 1943.0 |
| 16 | IR 87707-108-B-B-B | 82 | 89 | 95 | 80 | 6016.2 | 1974.8 |
| 17 | IR 87706-215-B-B-B | 81 | 86 | 95 | 75 | 5318.6 | 2366.3 |
| 18 | IR 87707-300-B-B-B | 80 | 86 | 92 | 74 | 5449.6 | 2774.6 |
| 19 | IR 87707-405-B-B-B | 80 | 88 | 95 | 80 | 6401.9 | 2931.3 |
| 20 | IR 87707-359-B-B-B | 81 | 88 | 98 | 76 | 6361.1 | 2581.3 |
| 21 | IR 87707-182-B-B-B | 78 | 86 | 97 | 79 | 5225.9 | 2891.6 |
| 22 | IR 87706-342-B-B-B | 81 | 87 | 103 | 77 | 6000.0 | 2278.3 |
| 23 | IR 87706-341-B-B-B | 80 | 88 | 101 | 77 | 6801.4 | 3196.3 |
| 24 | IR 86790-557-B-B-B | 82 | 87 | 97 | 78 | 5975.4 | 2261.1 |
| 25 | IR 87706-224-B-B-B | 79 | 86 | 92 | 80 | 6522.0 | 2611.5 |
| 26 | IR 87707-56-B-B-B | 82 | 89 | 97 | 79 | 6104.1 | 1909.6 |
| 27 | IR 87707-312-B-B-B | 80 | 90 | 99 | 72 | 6814.9 | 1611.8 |
| 28 | IR 87705-77-13-B | 78 | 86 | 97 | 75 | 6135.5 | 2583.6 |
| 29 | IR 87705-20-12-B | 81 | 89 | 89 | 69 | 6058.8 | 1940.3 |
| 30 | IR 87705-17-6-B | 83 | 88 | 92 | 72 | 6144.7 | 1972.5 |
| 31 | IR 87705-21-13-B | 78 | 86 | 100 | 66 | 6224.8 | 1484.5 |
| 32 | IR 87705-6-9-B | 78 | 86 | 87 | 82 | 6107.0 | 2347.1 |
| 33 | IR 87705-6-8-B | 81 | 85 | 88 | 75 | 6207.6 | 2588.4 |
| 34 | IR 87705-85-4-B | 81 | 89 | 93 | 71 | 6459.2 | 1906.1 |
| 35 | IR 87705-80-15-B | 81 | 88 | 89 | 74 | 5515.9 | 2151.0 |
| 36 | IR 87705-36-3-B | 82 | 88 | 97 | 74 | 6909.3 | 2116.4 |
| 37 | IR 87705-17-5-B | 81 | 87 | 92 | 74 | 6221.8 | 1919.2 |
| 38 | IR 87705-58-5-B | 78 | 84 | 88 | 70 | 5516.8 | 2609.0 |
| 39 | IR 87705-81-14-B | 77 | 86 | 94 | 78 | 6191.1 | 2061.2 |
| 40 | IR 87705-16-8-B | 81 | 88 | 94 | 71 | 5983.1 | 2416.4 |
| 41 | IR 87705-15-9-B | 79 | 87 | 92 | 74 | 5642.7 | 2037.6 |
| 42 | IR 87705-7-15-B | 80 | 87 | 97 | 75 | 6543.6 | 2280.3 |
| 43 | IR 87705-10-11-B | 79 | 88 | 93 | 78 | 6032.3 | 2321.0 |
| 44 | IR 87705-49-9-B | 79 | 86 | 97 | 76 | 6454.1 | 1970.5 |
| 45 | IR 87705-44-4-B | 80 | 87 | 98 | 72 | 6175.5 | 2000.4 |
| 46 | IR 87705-19-8-B | 82 | 89 | 96 | 73 | 5675.9 | 2006.4 |
| 47 | IR 87705-14-11-B | 77 | 87 | 95 | 76 | 6055.4 | 2283.4 |
| 48 | IR 87705-83-12-B | 80 | 88 | 95 | 72 | 5526.3 | 2269.7 |
| 49 | IR 87705-31-3-B | 84 | 90 | 89 | 68 | 6429.9 | 1699.7 |
| 50 | IR 87705-25-4-B | 82 | 89 | 94 | 75 | 6348.8 | 2479.8 |
| 51 | IR 87705-42-6-B | 79 | 85 | 93 | 76 | 6298.6 | 2429.6 |
| 52 | IR 87705-72-12-B | 80 | 87 | 91 | 75 | 6090.0 | 1892.4 |
| 53 | IR 87728-395-B-B | 83 | 89 | 92 | 70 | 6626.6 | 2045.6 |
| 54 | IR 87728-102-B-B | 83 | 91 | 89 | 75 | 6733.5 | 1534.0 |
| 55 | IR 87728-30-B-B | 80 | 89 | 93 | 76 | 6434.7 | 2203.7 |
| 56 | IR 87728-200-B-B | 83 | 91 | 91 | 77 | 6408.9 | 1292.5 |
| 57 | IR 87728-466-B-B | 80 | 90 | 93 | 73 | 5581.8 | 1641.1 |
| 58 | IR 87728-23-B-B | 81 | 89 | 94 | 73 | 6822.8 | 1947.6 |
| 59 | IR 87728-162-B-B | 84 | 91 | 94 | 75 | 6114.6 | 1636.4 |
| 60 | IR 87728-26-B-B | 83 | 89 | 95 | 73 | 6843.5 | 2173.6 |
| 61 | IR 87728-520-B-B | 83 | 90 | 93 | 72 | 6541.1 | 1244.5 |
| 62 | IR 87728-513-B-B | 84 | 92 | 90 | 72 | 7049.8 | 1434.9 |
| 63 | IR 87728-59-B-B | 81 | 89 | 90 | 77 | 6601.7 | 1515.0 |
| 64 | IR 87728-367-B-B | 79 | 91 | 86 | 69 | 6416.7 | 1221.5 |
| 65 | IR 87728-409-B-B | 81 | 89 | 96 | 70 | 6572.2 | 2112.8 |
| 66 | IR 87728-491-B-B | 82 | 89 | 95 | 72 | 6232.4 | 1879.2 |
| 67 | IR 87728-94-B-B | 81 | 90 | 89 | 72 | 6206.9 | 1736.5 |
| 68 | IR 87728-75-B-B | 84 | 90 | 93 | 69 | 6736.3 | 1840.4 |
| 69 | IR 84170-1573-2-B-B-1 | 77 | 80 | 99 | 86 | 5433.4 | 2293.8 |
| 70 | IR 84170-1655-2-B-B-1 | 78 | 84 | 93 | 78 | 5082.9 | 2300.6 |
| 71 | IR 84170-1677-2-B-B-4 | 82 | 90 | 97 | 80 | 6720.8 | 1730.8 |
| 72 | IR 84170-1692-2-B-B-1 | 80 | 88 | 96 | 75 | 5541.7 | 1439.1 |
| 73 | IR 84170-1424-B-B-1 | 79 | 86 | 91 | 77 | 5542.7 | 2277.3 |
| 74 | IR 84170-1457-B-B-1 | 79 | 88 | 93 | 75 | 5619.0 | 1438.3 |
| 75 | IR 84170-1474-B-B-1 | 80 | 90 | 94 | 71 | 5673.8 | 1665.1 |
| 76 | IR 84170-1537-B-B-B | 79 | 88 | 90 | 75 | 5852.9 | 1552.2 |
| 77 | IR 84170-1541-B-B-1 | 80 | 85 | 98 | 80 | 5689.4 | 2265.7 |
| 78 | IR 84170-1573-B-B-1 | 78 | 85 | 100 | 77 | 6149.4 | 2987.1 |
| 79 | IR 84170-1644-B-B-1 | 79 | 87 | 97 | 80 | 6167.8 | 1797.0 |
| 80 | IR 84170-1721-B-B-1 | 84 | 91 | 93 | 72 | 6603.9 | 1559.6 |
| 81 | IR 84170-1733-B-B-1 | 79 | 86 | 89 | 79 | 4767.8 | 2784.9 |
| 82 | IR 84170-1733-B-B-2 | 77 | 84 | 92 | 79 | 4986.5 | 3069.6 |
| 83 | IR 84170-1733-B-B-3 | 78 | 80 | 90 | 82 | 5124.6 | 2819.6 |
| 84 | IR 84170-1757-B-B-1 | 81 | 88 | 93 | 70 | 6321.5 | 2430.6 |
| 85 | IR 64 | 80 | 88 | 92 | 76 | 5435.2 | 1438.6 |
| 86 | Aday Sel | 77 | 77 | 135 | 100 | 4658.0 | 1036.1 |

DTF: days to flowering, HTAVE: average plant height, GYKGPHA: grain yield (kg ha^-1^)
