# Supplementary material for: Genetic, Physiological, and Gene Expression Analyses Reveal That Multiple QTL Enhance Yield of Rice Mega-Variety IR64 under Drought
Source: PLoS One. 2013 May 8;8(5):e62795. doi: 10.1371/journal.pone.0062795 (PMC3648568; doi:10.1371/journal.pone.0062795)
Supplement: Table S7 — List of candidate genes associated with yield-under-drought QTLs. (DOCX) [file pone.0062795.s010.docx]

**Table S7.**

| Gene ID (MSU annotation) | Description |
| --- | --- |
| OS02G12900 | cysteine synthase, putative, expressed |
| OS02G12939 | expressed protein |
| OS02G13020 | expressed protein |
| OS02G13060 | expressed protein |
| OS02G13090 | hypothetical protein |
| OS02G13100 | catalytic/protein phosphatase type 2C, putative, expressed |
| OS02G13140 | xaa-Pro dipeptidase, putative, expressed |
| OS02G13160 | ATPP2-A13, putative, expressed |
| OS02G13170 | mitochondrial carrier protein, expressed |
| OS02G13220 | expressed protein |
| OS02G13304 | expressed protein |
| OS02G13450 | hypothetical protein |
| OS02G13570 | ATP-binding protein, putative, expressed |
| OS04G12620 | hypothetical protein |
| OS04G12678 | indole-3-acetate beta-glucosyltransferase, putative |
| OS04G12690 | indole-3-acetate beta-glucosyltransferase, putative |
| OS04G12960 | indole-3-acetate beta-glucosyltransferase, putative, expressed |
| OS04G12970 | indole-3-acetate beta-glucosyltransferase, putative, expressed |
| OS09G24580 | calmodulin-like protein, putative, expressed |
| OS09G24640 | selenium-binding protein, putative, expressed |
| OS09G24650 | protein-binding protein, putative, expressed |
| OS09G24660 | zinc finger motif, C2HC5-type family protein, putative, expressed |
| OS09G24670 | CAAX amino terminal protease family protein, putative, expressed |
| OS09G24800 | myb-related protein Myb4, putative |
| OS09G24910 | 6-phosphofructokinase 2, putative, expressed |
| OS09G24954 | double-stranded RNA-binding motif family protein, expressed |
| OS09G24980 | golgi SNARE 12 protein, putative, expressed |
| OS09G25050 | EMB2745, putative, expressed |
| OS09G25150 | dihydroflavonol-4-reductase, putative, expressed |
| OS09G25190 | ubiquitin-protein ligase/zinc ion-binding protein, putative, expressed |
| OS09G25310 | 26S proteasome non-ATPase regulatory subunit 9, putative, expressed |
| OS09G25314 | cytochrome c oxidase copper chaperone, putative, expressed |
| OS09G25370 | deoxyhypusine synthase, putative, expressed |
| OS09G25410 | expressed protein |
| OS09G25420 | zinc finger, C2H2-type family protein, expressed |
| OS09G25460 | anthranilate N-benzoyltransferase protein 1, putative, expressed |
| OS09G25470 | hypothetical protein |
| OS09G25550 | expressed protein |
| OS09G25610 | defense-related protein, putative, expressed |
| OS09G25620 | S-adenosylmethionine decarboxylase proenzyme, putative, expressed |
| OS09G25720 | acanthoscurrin-1 precursor, putative, expressed |
| OS09G25740 | expressed protein |
| OS09G25760 | senescence-associated protein DH, putative, expressed |
| OS09G25784 | nodulin-like protein 5NG4, putative, expressed |
| OS09G25880 | expressed protein |
| OS09G25910 | basic 7S globulin precursor, putative |
| OS09G25934 | expressed protein |
| OS09G25950 | uncharacterized ACR, putative, expressed |
| OS09G25970 | hypothetical protein |
| OS09G26004 | expressed protein |
| OS09G26180 | transcription initiation factor TFIID subunit 10, putative, expressed |
| OS09G26190 | CBS domain-containing protein, expressed |
| OS09G26260 | ATP-binding protein, putative, expressed |
| OS09G26300 | hypro1, putative, expressed |
| OS09G26310 | hypro1, putative, expressed |
| OS09G26320 | hypro1, putative |
| OS09G26340 | histone H4, putative, expressed |
| OS09G26350 | expressed protein |
| OS09G26400 | protein-binding protein, putative, expressed |
| OS09G26430 | retrotransposon protein, putative, unclassified |
| OS09G26440 | expressed protein |
| OS09G26500 | serine hydrolase, putative, expressed |
| OS09G26520 | hypothetical protein |
| OS09G26530 | expressed protein |
| OS09G26540 | expressed protein |
| OS09G26550 | protease Do-like 14, putative, expressed |
| OS09G26554 | expressed protein |
| OS09G26560 | expressed protein |
| OS09G26960 | flavonoid 3-monooxygenase, putative, expressed |
| OS10G35090 | rf1 protein, mitochondrial precursor, putative, expressed |
| OS10G35230 | rf1 protein, mitochondrial precursor, putative, expressed |
| OS10G35240 | rf1 protein, mitochondrial precursor, putative, expressed |
| OS10G35260 | rf1 protein, mitochondrial precursor, putative |
| OS10G35294 | fiber protein Fb34, putative, expressed |
| OS10G35436 | rf1 protein, mitochondrial precursor, putative, expressed |
| OS10G35440 | rf1 protein, mitochondrial precursor, putative, expressed |
| OS10G35500 | epoxide hydrolase, putative, expressed |
| OS10G35520 | epoxide hydrolase 2, putative, expressed |
| OS10G35530 | epoxide hydrolase 2, putative, expressed |
| OS10G35560 | expressed protein |
| OS10G35630 | expressed protein |
| OS10G35650 | expressed protein |
| OS10G35690 | ribosomal protein S18-containing protein, expressed |
| OS10G35720 | OsGrx_S17 - glutaredoxin subgroup II, expressed |
| OS10G35730 | expressed protein |
| OS10G35800 | expressed protein |
| OS10G35820 | expressed protein |
| OS10G35990 | ATP-dependent RNA helicase dhh1, putative, expressed |
| OS10G36000 | remorin, putative, expressed |
| OS10G36070 | nonspecific lipid-transfer protein precursor, putative |
| OS10G36090 | nonspecific lipid-transfer protein precursor, putative |
| OS10G36100 | nonspecific lipid-transfer protein precursor, putative, expressed |
| OS10G36170 | nonspecific lipid-transfer protein precursor, putative, expressed |
| OS10G36210 | valyl-tRNA synthetase, putative |
| OS10G36250 | TPR domain-containing protein, expressed |
| OS10G36270 | disease resistance RPP13-like protein 1, putative, expressed |
