# Supplementary material for: Genetic, Physiological, and Gene Expression Analyses Reveal That Multiple QTL Enhance Yield of Rice Mega-Variety IR64 under Drought
Source: PLoS One. 2013 May 8;8(5):e62795. doi: 10.1371/journal.pone.0062795 (PMC3648568; doi:10.1371/journal.pone.0062795)
Supplement: Table S8 — Candidate genes/gene families and their biological functions under stress conditions. (DOCX) [file pone.0062795.s011.docx]

**Table S8.**

| **Gene ID**  **(MSU annotation)** | **Description** | **Biological function of the gene/gene family** | **Reference** |
| --- | --- | --- | --- |
| OS02G12900 | cysteine synthase, putative, expressed | tolerance of metal toxicity, aluminum stress tolerance in rice | 1 |
| OS02G13100 | catalytic/protein phosphatase type 2C, putative, expressed | abiotic stress signaling | 2 |
| OS02G13140 | xaa-Pro dipeptidase, putative, expressed | regulatory function involved in plant defense mechanism, plant fertility | 3 |
| OS02G13170 | mitochondrial carrier protein, expressed | anaerobic seed germination | 4 |
| OS02G13570 | ATP-binding protein, putative, expressed | drought stress response | 5 |
| OS04G12678 | indole-3-acetate beta-glucosyltransferase, putative | drought, salt, and cold stress responses | 6 |
| OS09G24580 | calmodulin-like protein, putative, expressed | signaling and stress tolerance | 7 |
| OS09G24640 | selenium-binding protein, putative, expressed | abiotic stress and mineral toxicity stress tolerance | 8, 9 |
| OS09G24650 | protein-binding protein, putative, expressed | regulatory protein | _ |
| OS09G24660 | zinc finger motif, C2HC5-type family protein, putative, expressed | stress responsive | _ |
| OS09G24670 | CAAX amino terminal protease family protein, putative, expressed | proteolytic cleavage and removal of cargo bodies from the cell | _ |
| OS09G24800 | myb-related protein Myb4, putative | adaptive response to drought and cold | 10 |
| OS09G24910 | 6-phosphofructokinase 2, putative, expressed | key enzyme in glycolysis and respiration | 11 |
| OS09G24954 | double-stranded RNA-binding motif family protein, expressed | growth, development, and stress response | 12 |
| OS09G24980 | golgi SNARE 12 protein, putative, expressed | involved in stress signaling and development | 13 |
| OS09G25050 | EMB2745, putative, expressed | embryo growth and development | _ |
| OS09G25150 | dihydroflavonol-4-reductase, putative, expressed | growth, development, and stress response | 14, 15 |
| OS09G25190 | ubiquitin-protein ligase | hormonal signaling, growth, and development | 16 |
| OS09G25310 | 26S proteasome non-ATPase regulatory subunit 9, putative, expressed | involved in plant developmental process | 17 |
| OS09G25314 | cytochrome c oxidase copper chaperone, putative, expressed | stress inducible, active in meristematic tissue | 18 |
| OS09G25370 | deoxyhypusine synthase, putative, expressed | delayed senescence induced by drought | 19 |
| OS09G25420 | zinc finger, C2H2-type family protein, expressed | stress tolerance | 20 |
| OS09G25460 | anthranilate N-benzoyltransferase protein 1, putative, expressed | adaptation to salinity stress | 21 |
| OS09G25610 | defense-related protein, putative, expressed | defense against biotic and abiotic stresses | _ |
| OS09G25620 | S-adenosylmethionine decarboxylase proenzyme, putative, expressed | salt and drought stress response | 22 |
| OS09G25720 | acanthoscurrin-1 precursor, putative, expressed | stress induced | _ |
| OS09G25760 | senescence-associated proteins | biotic and abiotic stress responsive | 23 |
| OS09G25784 | nodulin-like protein 5NG4, putative, expressed | lateral or adventitious root formation | 24 |
| OS09G25910 | basic 7S globulin precursor, putative | abiotic stress tolerance | 25 |
| OS09G25950 | uncharacterized ACR, putative, expressed | signaling and environmental response | 26 |
| OS09G26180 | transcription initiation factor TFIID subunit 10, putative, expressed | regulatory role | _ |
| OS09G26190 | CBS domain-containing protein, expressed | involved in signaling pathways of biotic and abiotic stress | 27 |
| OS09G26300 | hypro1, putative, expressed | stigma-specific protein | 28 |
| OS09G26340 | histone H4, putative, expressed | stress-inducible changes, regulation of gene expression | 29 |
| OS09G26430 | retrotransposon protein, putative, unclassified | _ | _ |
| OS09G26500 | serine hydrolase, putative, expressed | drought response | 30 |
| OS09G26550 | protease Do-like 14, putative, expressed | proteolytic cleavage and removal of cargo bodies from the cell | _ |
| OS09G26960 | flavonoid 3-monooxygenase, putative, expressed | growth, development, and stress response | 14, 15 |
| OS10G35090 | rf1 protein, mitochondrial precursor, putative, expressed | fertility restoration | 31, 32 |
| OS10G35294 | fiber protein Fb34, putative, expressed | stress-responsive gene, secondary cell wall formation, xylem production | 33 |
| OS10G35500 | epoxide hydrolase, putative, expressed | drought response | IRRI book |
| OS10G35690 | ribosomal protein S18-containing protein, expressed | meristematic tissue activity and plant development | 34 |
| OS10G35720 | OsGrx_S17 - glutaredoxin subgroup II, expressed | oxidative stress response | 35 |
| OS10G35990 | ATP-dependent RNA helicase dhh1, putative, expressed | involved in abiotic stress tolerance | 36 |
| OS10G36000 | remorin, putative, expressed | response to biotic and abiotic stresses, signal transduction | 37 |
| OS10G36070 | nonspecific lipid-transfer protein precursor, putative | involved in signaling pathways | 38 |
| OS10G36210 | valyl-tRNA synthetase, putative | heat stress induced | 39 |
| OS10G36250 | TPR domain-containing protein, expressed | osmotic stress response and abscisic sensitivity, seed development | 40 |
| OS10G36270 | disease resistance RPP13-like protein 1, putative, expressed | involved in insect and disease resistance | 41 |
