# Supplementary material for: Genetic, Physiological, and Gene Expression Analyses Reveal That Multiple QTL Enhance Yield of Rice Mega-Variety IR64 under Drought
Source: PLoS One. 2013 May 8;8(5):e62795. doi: 10.1371/journal.pone.0062795 (PMC3648568; doi:10.1371/journal.pone.0062795)
Supplement: Text S1 — Supplementary References. (DOCX) [file pone.0062795.s012.docx]

**Text S1.**

1. Yang Q, Wang Y, Zhang J, Shi W, Qian C, et al. (2007) Identification of aluminum-responsive proteins in rice roots by a proteomic approach: cysteine synthase as a key player in Al response. Proteomics 7:737–749.
2. Xiong L, Schumaker KS, Zhu JK (2002) Cell signaling during cold, drought, and salt stress. Plant Cell 14:S165–S183.
3. Walling LL (2006) Recycling or regulation? The role of amino-terminal modifying enzymes. Curr Opin Plant Biol 9:227–233.
4. Taylor NL, Howell KA, Heazlewood J, Yien T, Tan W, et al. (2010) Analysis of the rice mitochondrial carrier family reveals anaerobic accumulation of a basic amino acid carrier involved in arginine metabolism during seed germination. Plant Physiol 154:691–704.
5. Isokephi RD, Simmons SS, Cohly HHP, Ekunwe SIN, Begonia GB,  [(2011)](http://www.ncbi.nlm.nih.gov/sites/entrez?cmd=search&db=PubMed&term=%20Isokpehi%2BRD%5bauth%5d) Identification of drought-responsive universal stress proteins in *Viridiplantae*. Bioinformatics and Biol Insights 5:41–58.
6. Seki M, Narusaka M, Ishida J, Nanjo T, Fujita M, et al. (2002) Monitoring expression profiles of 7000 *Arabidopsis* genes under drought, cold and high-salinity stresses using a full-length cDNA microarray. Plant J 31:279–292.
7. [Xu GY](http://www.ncbi.nlm.nih.gov/pubmed?term=Xu%20GY%5BAuthor%5D&cauthor=true&cauthor_uid=21359958), Rocha PS, Wang ML, Xu ML, Cui YC, et al. (2011) A novel rice calmodulin-like gene, *OsMSR2*, enhances drought and salt tolerance and increases ABA sensitivity in *Arabidopsis*. [Planta](http://www.ncbi.nlm.nih.gov/pubmed/21359958) 234:47–59.
8. Liu X, Baird W (2003) Differential expression of genes regulated in response to drought or salinity stress in sunflower. Crop Sci 43:678-687.
9. Agalou A, Roussis A, Spaink HP (2005) The *Arabidopsis* selenium-binding protein confers tolerance to toxic levels of selenium. Funct Plant Biol 32:881–890.
10. [Pasquali G](http://www.ncbi.nlm.nih.gov/pubmed?term=Pasquali%20G%5BAuthor%5D&cauthor=true&cauthor_uid=18679687), [Biricolti S](http://www.ncbi.nlm.nih.gov/pubmed?term=Biricolti%20S%5BAuthor%5D&cauthor=true&cauthor_uid=18679687), [Locatelli F](http://www.ncbi.nlm.nih.gov/pubmed?term=Locatelli%20F%5BAuthor%5D&cauthor=true&cauthor_uid=18679687), [Baldoni E](http://www.ncbi.nlm.nih.gov/pubmed?term=Baldoni%20E%5BAuthor%5D&cauthor=true&cauthor_uid=18679687), [Mattana M](http://www.ncbi.nlm.nih.gov/pubmed?term=Mattana%20M%5BAuthor%5D&cauthor=true&cauthor_uid=18679687) (2008) *Osmyb4* expression improves adaptive responses to drought and cold stress in transgenic apples. [Plant Cell Rep](http://www.ncbi.nlm.nih.gov/pubmed/18679687##)orts 27:1677–1686.
11. Carnal NW, Black CC (1983) Phosphofructokinase activities in photosynthetic organisms. Plant Physiol 73:150–155.
12. Lorkovic ZJ (2009). Role of plant RNA-binding proteins in development, stress response and genome organization. Trends Plant Sci 14:229–236.
13. Bassham DC, Blatt MR (2008) SNAREs: cogs and coordinators in signaling and development. Plant Physiol 147:1504–1515.
14. Shirley WB (2011) Flavonoid biosynthesis: a colorful model for genetics, biochemistry, cell biology and biotechnology. Plant Physiol 126:485–493.
15. Londhe JS, Devasagayam TP, Foo LY, Ghaskadbi SS (2009) Radioprotective properties of polyphenols from *Phyllanthus amarus* linn. J Radiation Res 50:303–309.
16. Stone SL, Callis J (2007) Ubiquitin ligases mediate growth and development by promoting protein death. Curr Opin Plant Biol 10:624–632.
17. Sullivan JA, Shirasu K, Deng XW (2003) The diverse roles of ubiquitinine and the 26S proteasome in the life of plants. Nature 4:948–958.
18. Attallah CV, Welchen E, Gonzalez DH (2007) The promoters of *Arabidopsis thaliana* genes *AtCOX17-1* and *-2*, encoding a copper chaperone involved in cytochrome c oxidase biogenesis, are preferentially active in roots and anthers and induced by biotic and abiotic stress. Physiol Plantarum 129:123–134.
19. Wang TW, Lu L, Zhang CG, Taylor C, Thompson JE (2003) Pleiotropic effects of suppressing deoxyhypusine synthase expression in *Arabidopsis thaliana*. Plant Mol Biol 52:1223–1235.
20. [Agarwal P](http://www.ncbi.nlm.nih.gov/pubmed?term=Agarwal%20P%5BAuthor%5D&cauthor=true&cauthor_uid=17610133), Arora R, Ray S, Singh AK, Singh VP, et al. (2007) Genome-wide identification of C_2_H_2_ zinc-finger gene family in rice and their phylogeny and expression analysis. Plant Mol Biol65:467–485.
21. [Walia H](http://www.ncbi.nlm.nih.gov/pubmed?term=Walia%20H%5BAuthor%5D&cauthor=true&cauthor_uid=17324228), [Wilson C](http://www.ncbi.nlm.nih.gov/pubmed?term=Wilson%20C%5BAuthor%5D&cauthor=true&cauthor_uid=17324228), [Condamine P](http://www.ncbi.nlm.nih.gov/pubmed?term=Condamine%20P%5BAuthor%5D&cauthor=true&cauthor_uid=17324228), [Liu X](http://www.ncbi.nlm.nih.gov/pubmed?term=Liu%20X%5BAuthor%5D&cauthor=true&cauthor_uid=17324228), [Ismail AM](http://www.ncbi.nlm.nih.gov/pubmed?term=Ismail%20AM%5BAuthor%5D&cauthor=true&cauthor_uid=17324228), et al.(2007) Large-scale expression profiling and physiological characterization of jasmonic acid-mediated adaptation of barley to salinity stress. PlantCell Environ 30:410–421.
22. Li ZY, Chen SY (2000) Differential accumulation of the S-adenosylmethionine decarboxylase transcript in rice seedlings in response to salt and drought stresses. Theor Appl Genet 100:782–788.
23. [Degenkolbe](http://www.ncbi.nlm.nih.gov/sites/entrez?cmd=search&db=PubMed&term=%20Degenkolbe%2BT%5bauth%5d) T, Do PT, Zuther E, Repsilbder D, Walther D, et al. (2009) Expression profiling of rice cultivars differing in their tolerance to long-term drought stress. Plant Mol Biol 69:133–153.
24. Busov VB, Johannes E, Whetten RW, Sederoff RR, Spiker SL, et al. (2004) An auxin-inducible gene from loblolly pine (*Pinus taeda* L.) is differentially expressed in mature and juvenile-phase shoots and encodes a putative transmembrane protein. Planta 218:916–927.
25. Yoshizawa T, Hashimoto H, Shimizu T, Yamabe M, Shichijo N, et al. (2011) Purification, crystallization and X-ray diffraction study of basic 7S globulin from soybean. [Acta Crystallographica](http://www.chemeurope.com/en/publications/journals/acta-crystallographica-section-f/) 67:87–89.
26. Hsieh M, Goodman HM (2012) Molecular characterizations of a novel gene family encoding ACT domain repeat proteins in *Arabidopsis*. Plant Physiol 130:1797–1806.
27. Min WX, Hao F, Fei SY, Bo L, Jie WX, et al. (2010) Cloning and expression analysis of a CBS domain containing protein gene *TaCDCP1* from wheat. [Acta Agronomica Sin](http://211.155.251.148:8080/zwxb)ica 36:2091–2098.
28. Li M, Xu W, Yang W, Kong Z, Xue Y (2007) Genome-wide expression profiling reveals conserved and novel molecular functions of the stigma in rice (*Oryza sativa* L.). Plant Physiol 144:1797–1812.
29. Pawlak S, Deckert J (2007) Histone modifications under environmental stress. Biol Letters 44:65-73.
30. Lenka SK, Katiyar A, Chinnusamy V, Bansal KC (2011) Comparative analysis of drought-responsive transcriptome in indica rice genotypes with contrasting drought tolerance. Plant Biotech J  [9:](http://onlinelibrary.wiley.com/doi/10.1111/pbi.2011.9.issue-3/issuetoc)315–327.
31. Akagi H, Nakamura A, Yokozeki-Misono Y, Inagaki A, Takahashi H, et al. (2004) Positional cloning of the rice *Rf-1* gene, a restorer of BT-type cytoplasmic male sterility that encodes a mitochondria-targeting PPR protein. Theor Appl Genet108:1449–1457.
32. Wang Z, Zou Y, Li X, Zhang Q, Chen L, et al. (2006) Cytoplasmic male sterility of rice with Boro II cytoplasm is caused by a cytotoxic peptide and is restored by two related PPR motif genes via distinct modes of mRNA silencing. Plant Cell 18:676–687.
33. Ubeda-Tomas S (2007) Genomic-assisted identification of genes involved in secondary growth in *Arabidopsis* utilizing transcript profiling of poplar wood-forming tissues. Physiol Plantarum 129:415–428.
34. Lijsebettens MV, Vanderhaeghen R, Block MD, Bauw G, Villarroel R, et al. (1994) An S18 ribosomal protein gene copy at the *Arabidopsis* PFL locus affects plant development by its specific expression in meristems. The EMBO J13:3378–3388.
35. Cheng NH, Liu JZ, Liu X, Wu Q, Thomspon SM, et al. (2011) *Arabidopsis* monothiol glutaredoxin, *AtGRXS17*, is critical for temperature-dependent postembryonic growth and development via modulating auxin response. J Biol Chem286:20398–20406.
36. [Owttrim](http://nar.oxfordjournals.org/search?author1=George+W.+Owttrim&sortspec=date&submit=Submit) GW (2006) RNA helicases and abiotic stress. Nucleic Acids Res 34:3220–3230.
37. Raffaele S, Mongrand S, Gamas P, Niebel A, Ott T, et al. (2007) Genome-wide annotation of remorins, a plant-specific protein family: evolutionary and functional perspectives. Plant Physiol 145:593–600.
38. [Cheng](http://www.ncbi.nlm.nih.gov/sites/entrez?cmd=search&db=PubMed&term=%20Cheng%2BHC%5bauth%5d) HC,  [Cheng](http://www.ncbi.nlm.nih.gov/sites/entrez?cmd=search&db=PubMed&term=%20Cheng%2BPT%5bauth%5d) PT, [Peng](http://www.ncbi.nlm.nih.gov/sites/entrez?cmd=search&db=PubMed&term=%20Peng%2BP%5bauth%5d) P,  [Lyu](http://www.ncbi.nlm.nih.gov/sites/entrez?cmd=search&db=PubMed&term=%20Lyu%2BPC%5bauth%5d) PC,  [Sun](http://www.ncbi.nlm.nih.gov/sites/entrez?cmd=search&db=PubMed&term=%20Sun%2BYJ%5bauth%5d) YJ (2004) Lipid binding in rice nonspecific lipid transfer protein-1 complexes from *Oryza sativa.* [Protein Sci](http://www.ncbi.nlm.nih.gov/pubmed/15295114##) 3:2304–2315.
39. Qin D, Wu H, Peng H, Yao Y, Ni Z, et al. (2008) Heat stress-responsive transcriptome analysis in heat susceptible and tolerant wheat (*Triticum aestivum* L.) by using wheat genome array. BMC Genomics 9:432.
40. Rosado A, Schapire AL, Bressan RA, Harfouche AL, Hasegawa PM, et al. (2006) The *Arabidopsis* tetratricopeptide repeat–containing protein TTL1 is required for osmotic stress responses and abscisic acid sensitivity. Plant Physiol 42:1113–1126.
41. Dubey M, Chandel G (2010) In silico survey and characterization of resistance gene analogues (RGAs) in the genomic regions encompassing gall midge resistance genes *Gm4* and *Gm5* in rice (*Oryza sativa* L.). Plant OMICS J 3:40–148.
